# Using syndromic measures of mortality to capture the dynamics of COVID-19 in Java, Indonesia, in the context of vaccination rollout

**DOI:** 10.1186/s12916-021-02016-2

**Published:** 2021-06-18

**Authors:** Bimandra A. Djaafara, Charles Whittaker, Oliver J. Watson, Robert Verity, Nicholas F. Brazeau, Widyastuti, Dwi Oktavia, Verry Adrian, Ngabila Salama, Sangeeta Bhatia, Pierre Nouvellet, Ellie Sherrard-Smith, Thomas S. Churcher, Henry Surendra, Rosa N. Lina, Lenny L. Ekawati, Karina D. Lestari, Adhi Andrianto, Guy Thwaites, J. Kevin Baird, Azra C. Ghani, Iqbal R. F. Elyazar, Patrick G. T. Walker

**Affiliations:** 1 MRC Centre for Global Infectious Disease Analysis and the Abdul Latif Jameel Institute for Disease and Emergency Analytics, School of Public Health, Imperial College London St Mary’s Campus, Norfolk Place, London, W2 1PG UK; 2 Eijkman-Oxford Clinical Research Unit, Jakarta, Indonesia; 3 Jakarta Provincial Department of Health, Jakarta, Indonesia; 4 School of Life Sciences, University of Sussex, Brighton, UK; 5 Centre for Tropical Medicine, Faculty of Medicine, Public Health and Nursing, Universitas Gadjah Mada, Yogyakarta, Indonesia; 6 Oxford University Clinical Research Unit, Ho Chi Minh City, Vietnam; 7 Centre for Tropical Medicine and Global Health, Nuffield Department of Medicine, University of Oxford, Oxford, UK

**Keywords:** COVID-19, Modelling, Indonesia, Non-pharmaceutical interventions, Vaccinations, Surveillance

## Abstract

**Background:**

As in many countries, quantifying COVID-19 spread in Indonesia remains challenging due to testing limitations. In Java, non-pharmaceutical interventions (NPIs) were implemented throughout 2020. However, as a vaccination campaign launches, cases and deaths are rising across the island.

**Methods:**

We used modelling to explore the extent to which data on burials in Jakarta using strict COVID-19 protocols (C19P) provide additional insight into the transmissibility of the disease, epidemic trajectory, and the impact of NPIs. We assess how implementation of NPIs in early 2021 will shape the epidemic during the period of likely vaccine rollout.

**Results:**

C19P burial data in Jakarta suggest a death toll approximately 3.3 times higher than reported. Transmission estimates using these data suggest earlier, larger, and more sustained impact of NPIs. Measures to reduce sub-national spread, particularly during Ramadan, substantially mitigated spread to more vulnerable rural areas. Given current trajectory, daily cases and deaths are likely to increase in most regions as the vaccine is rolled out. Transmission may peak in early 2021 in Jakarta if current levels of control are maintained. However, relaxation of control measures is likely to lead to a subsequent resurgence in the absence of an effective vaccination campaign.

**Conclusions:**

Syndromic measures of mortality provide a more complete picture of COVID-19 severity upon which to base decision-making. The high potential impact of the vaccine in Java is attributable to reductions in transmission to date and dependent on these being maintained. Increases in control in the relatively short-term will likely yield large, synergistic increases in vaccine impact.

**Supplementary Information:**

The online version contains supplementary material available at 10.1186/s12916-021-02016-2.

## Background

As of 28 April 2021, Indonesia has reported the highest number of confirmed COVID-19 cases (1,657,035) and deaths (45,116) among Southeast Asian countries [1]. Cases were first reported in West Java province, on the island of Java, on 2 March 2020, amid concern that the disease had circulated widely before [2, 3]. The city of Jakarta (the capital of Indonesia) subsequently became the epicentre of the country’s epidemic, following which the disease spread throughout the island.

**Fig. 1:**
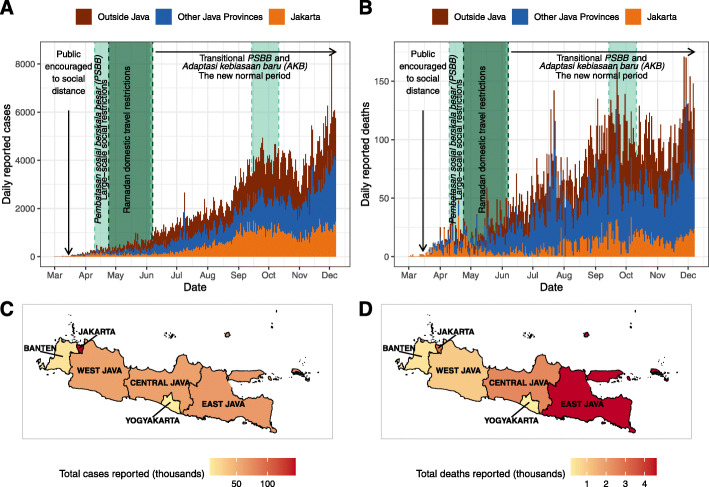
Burden of COVID-19 and timeline of interventions in Indonesia (data up to 7 December 2020). **a** Daily number of reported COVID-19 cases. **b** Daily number of reported COVID-19 deaths. **c** Total reported COVID-19 cases at province level in Java island. **d** Total reported COVID-19 deaths at province level in Java island

Non-pharmaceutical interventions (NPIs) have included national social distancing measures encouraging people to work, study, and worship at home (March 15) [4]; mandated social distancing measures implemented on April 10 as part of a lockdown, named *Pembatasan Sosial Berskala Besar* or PSBB in Indonesian [4]; and a ban on domestic travel during the month of Ramadan (April 24 to June 7) [5]. In June, Indonesia entered the *Adaptasi Kebiasaan Baru* (AKB or ‘new normal’) period where some restrictions were lifted (Fig. 1a and b) [4].

During this AKB period, the reported incidence of COVID-19 cases and deaths increased across Indonesia, with community transmission evident across the six provinces of Java (Fig. 1c and d). PSBB was subsequently reimposed in mid-September for 4 weeks in Jakarta in response to pressures on healthcare facilities across the city [6]. Cases and deaths continued to rise in early 2021, prompting further restrictions in districts across the island from January 11 [7]. On 13 January 2021, Indonesia initiated a nationwide vaccination campaign [7, 8]. The campaign initially involved vaccinating health care workers but was extended to the elderly and public workers on 17 February 2021 [9].

Understanding the trajectory of the epidemic in Java has been challenging. As in many countries [10, 11], testing constraints in Indonesia have limited the extent to which officially confirmed cases reflect underlying trends. Similar concerns exist for mortality data, based upon the high numbers of individuals exhibiting COVID-19 like symptoms who die before receiving a diagnosis [12, 13]. In Jakarta, such individuals are buried under strict COVID-19 protocols (C19P). Here, we use mathematical modelling approaches incorporating these data, and other measures of suspected mortality, to better understand the dynamics and burden of the epidemic experienced across Java to date, evaluate the impact of control measures, and understand how these past actions will shape future burden and vaccine impact.

## Methods

### Assessing SARS-CoV-2 transmissibility over time in Jakarta

Daily numbers of confirmed COVID-19 cases, deaths, and C19P funerals [14] were used to reconstruct daily incidence of symptom onset, using delay distributions between symptom onset and case reporting or death derived from individual patient data obtained from the Jakarta Department of Health (Additional file 1: Figure S1 [15–26]). For each data source (cases, deaths, and C19P funerals), 100 reconstructed time-series of daily incidence of symptom onset were generated, with estimates also adjusted for right-censoring in individuals where outcomes had yet to occur (Additional file 2: S2).

These reconstructed time series were translated into estimates of the daily effective reproduction number (*R*_*t*,*case*_ for cases, *R*_*t*,*death*_ for deaths, and *R*_*t*,*funeral*_ for funerals) in Jakarta using EpiEstim [27, 28]. This package estimates *R*_*t*_ using a branching process-based estimator that incorporates information on the serial interval distribution and dates of onsets of symptoms. Correlations between estimated *R*_*t*_ and the average daily changes in non-residential mobility [29] were assessed based on 1000 posterior samples from each estimated *R*_*t*_ time series and compared using Pearson’s correlation coefficient formula.

### Modelling subnational COVID-19 spread across Java

We developed a district-level metapopulation model to explore the expected spread of COVID-19 across the island of Java (Additional file 2: S5 [15–26]). For each district, stochastic differential equations representing a Susceptible-Exposed-Infected-Recovered (SEIR) model were implemented. Movement matrices were derived from anonymized mobile phone data, with separate matrices calculated for the high-migration period of Ramadan. Disease severity parameters were adjusted to account for the demography of each district. Transmissibility of the virus over time was calculated under the assumption that the relationship between mobility and *R*_*t*_ observed in Jakarta was informative across the rest of the island, exploring multiple assumptions about the transmissibility of COVID-19 in rural districts relative to urban districts (Additional file 1: Table S3 [15–26]). We simulated five different scenarios to assess the impact of restrictions (namely PSBB and Ramadan movement restrictions) on COVID-19 deaths and hospitalisation rates across Java (Table 1).

**Table 1:** Metapopulation model simulation scenarios (one baseline scenario and four counterfactual scenarios)

Scenario name	Details
Baseline	Movement from a district is assumed to reduce according to reductions in movement within a district scaled by an odds ratio of 2 to reflect assumed lower likelihood of travel outside a district relative to travel within a district.
Ramadan 1	No movement reductions between districts during the Ramadan and Eid festivals period and the *R*_*t*_ values during the period were assumed to be similar to the baseline scenario.
Ramadan 2	No movement reductions between districts during the Ramadan and Eid festivals period and the *R*_*t*_ values during the period were assumed to be 75% of each district *R*_0,*i*_.
Ramadan 3	No movement reductions between districts during the Ramadan and Eid festivals period and the *R*_*t*_ values during the period were assumed to be the same as each district *R*_0,*i*_.
Unmitigated	No interventions assumed which implies no movement reductions over all period of simulations with the *R*_*t*_ values to be the same as each district *R*_0,*i*_ over the period of simulations.

### Assessing the current province-level spread of the pandemic in Java and generating future scenarios

To estimate the recent trajectory of the epidemic and current cumulative levels of spread within each province, we adapted an existing modelling framework allowing the relationship between mobility and transmission to vary over time [10]. This allows us to capture the observed decoupling between aggregated movement patterns and burden in the ‘new normal’ period and simulate scenarios of future spread within each province. We fit this modelling framework both to officially reported COVID-19 deaths, as well as estimated suspected deaths, which include deaths of probable cases (i.e., patients with clinical criteria or chest imaging suggestive of COVID-19), which have been published by World Health Organization (WHO) Indonesia [4]. As the published suspected deaths data are only available on a weekly basis between 1 June to 29 November 2020, we augmented the data to reflect the entire time-period of the epidemic based upon the proportion of all suspected deaths (i.e., probable and confirmed) that were confirmed by each province in the period covered by the WHO reports (Additional file 2: S6 [15–26]).

Our future scenarios are projected based on a future ‘reproduction number under control’, *R*_*c*_, defined similarly to *R*_0_ as the average number of secondary infections within an entirely susceptible population but incorporating the impact of NPIs (and, equivalently, *R*_*t*_ but not incorporating the effects of population-level immunity such that *R*_0_ > *R*_*c*_ > *R*_*t*_). We evaluated three scenarios: a ‘current trajectory’ scenario (where the current trajectory of the epidemic continues with approximated *R*_*c*_ = 1.25), a ‘suppression’ scenario (where the transmission in the population is assumed to be immediately suppressed with *R*_*c*_ = 0.75) and an ‘unmitigated’ scenario (where the epidemic was assumed to be uncontrolled with *R*_*c*_ = 2.00). Our first set of projections were generated from September 2 onwards [30], at a time where policymakers were attempting to understand the potential benefits of the implementation of further NPIs, such as the reimposition of PSBB in Jakarta, which was then scheduled to be implemented on September 14 [6], in the context of no vaccine yet being any available. These scenarios evaluated the potential trajectory of the epidemic throughout 2021, including the impact of a ‘return-to-normal’ (*R*_*c*_ = 2.00) once burden had returned to low-levels (median of simulated trajectories reached less than 7 cumulative deaths over 7 days period)*.* Our current set of projections are generated from December 7 and in the context of an imminent vaccine campaign. Given the large remaining uncertainties in rollout and effectiveness, we do not incorporate any role of the vaccine. Instead, we aim to understand how different scenarios involving NPIs over the next few weeks and months will shape the potential longer-term effectiveness of future strategies in which vaccines will likely feature as a major component. To do this, we evaluate how both the number of lives lost, and number of lives that remain to be saved is likely to change incrementally by month according to the same future scenarios (i.e., current mitigation, suppression and unmitigated), relative to an unmitigated epidemic from the date of our projection (7 December 2020).

## Results

### Understanding initial establishment, transmission, and dynamics of SARS-CoV-2 in Jakarta

**Fig. 2:**
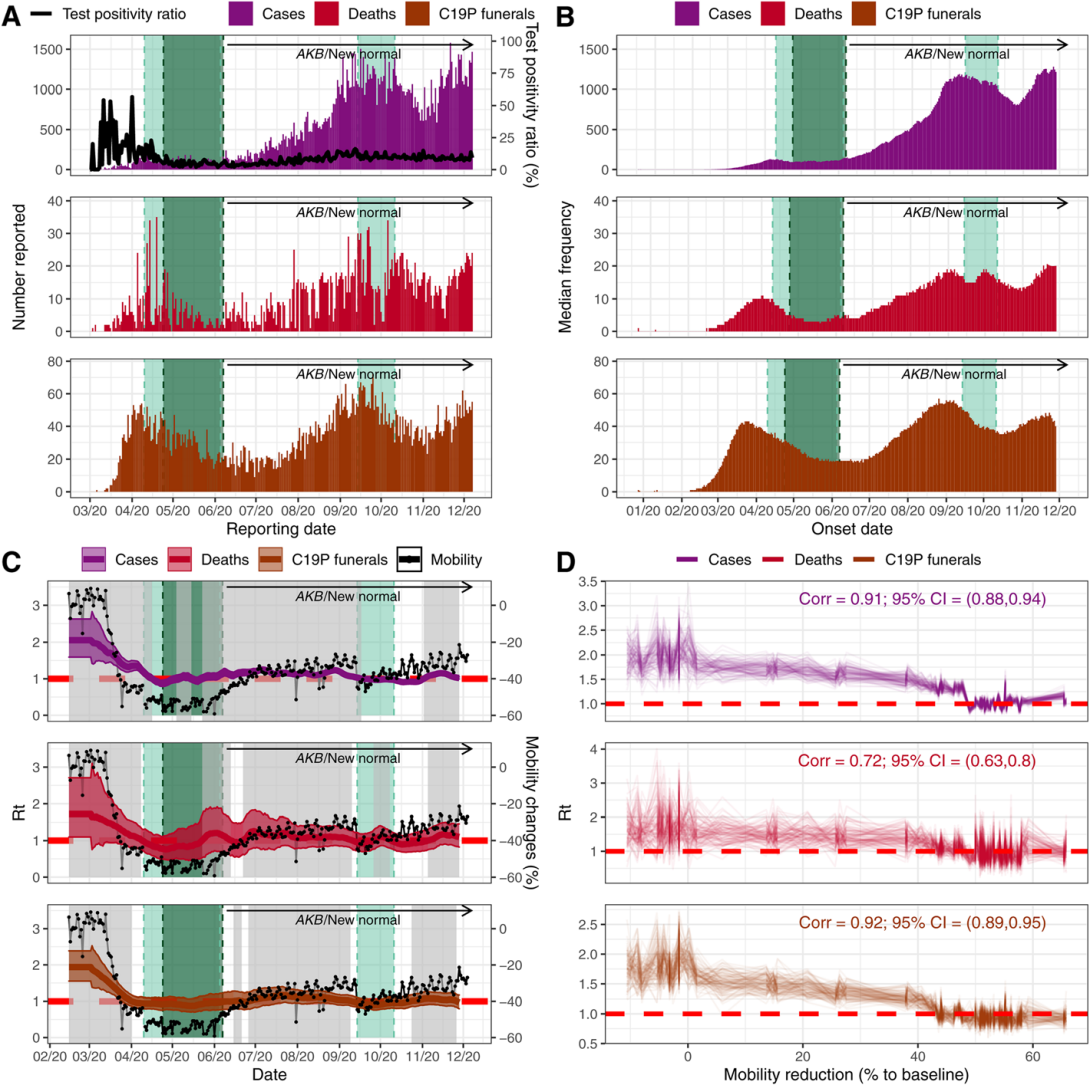
Temporal trends of cases, deaths, C19P funerals and respective estimates of R_t_ relative to the timing of intervention measures. Light green shaded areas denote periods of PSBB whilst the dark green shaded areas represent the period of Ramadan domestic travel restrictions. **a** Daily reported cases, deaths, and C19P funerals in Jakarta. Black line denotes the daily test positivity ratio. **b** Estimated frequency of symptom onset date of reported cases, deaths, and C19P funerals. Each bar represents the median daily frequency of 100 stochastic reconstructions. **c** Coloured lines and regions show, respectively, median and 95% CrI of estimated *R*_*t*_ (left y-axis) based on the reconstructed data (cases, deaths or C19P funerals). Grey areas denote periods where the estimated median *R*_*t*_ is above 1. Black lines and dots denote average changes in non-residential mobility (right y-axis). **d** The relationship and correlation coefficient between the estimated *R*_*t*_ and the average non-residential mobility reduction (up to 4 June 2020 or before the lifting of the first PSBB)

Figure 2a shows the daily reported cases, deaths, test positivity ratios, and funerals with C19P in Jakarta, transformed into inferred dates of symptom onset (Fig. 2b) using the relevant delay distributions. We estimate that 31 (22–41 95% CrI) and 124 (107–139 95% CrI) confirmed deaths and C19P funerals (assuming all funerals represent deaths due to COVID-19) had symptom onset occurring before 2nd March when COVID-19 was first identified in Indonesia. We estimate 10,950 (7530–14,040 95% CrI) infections based on confirmed deaths or 42,100 (36,280–47,570 95% CrI) based on C19P funerals (reflecting an assumption that all undiagnosed individuals provided with a C19P funeral would have tested positive) had occurred in Jakarta by March 2.

Reported cases in Jakarta appear to indicate two epidemic peaks to date (around mid-April and mid-September, when PSBB was imposed), with the number of cases reported during the second peak far higher than the first (Fig. 2b). However, the test-positivity rate declined in the first half of 2020, indicating increased testing rates and case-ascertainment, which complicates the interpretation of trends based on case data alone. Indeed, data on C19P funerals suggest that the first peak in infections likely occurred in mid-March and that infection levels during the second peak were at levels comparable to their initial peak.

Our branching-process-based estimates of *R*_*t*_ support the substantial impact of NPIs when applied to all three metrics (Fig. 2c). We estimate *R*_*t*_ to be between 1.5 and 2.5 initially, subsequently declining to below 1 during the first PSBB period, followed by a more recent increase to slightly above 1 as Jakarta entered the transitional PSBB in early June. The reimposition of the second PSBB in September also brought the *R*_*t*_ to below 1. Figure 2d shows a strong and significant correlation between *R*_*t*_ estimates with observed mobility patterns as measured by Google Mobility Reports (0.91, 0.72, and 0.92 for cases, deaths, and C19P funerals, respectively, all with p < 0.001) observed before the lifting of the first PSBB, though showing little correlation after the lifting of the first PSBB (Additional file 1: Figure S3 [15–26]). Estimates based upon funeral trends support a more rapid, larger, and more sustained impact of interventions than those based upon case reporting. The correlation with within-city mobility is lowest for the deaths data, where substantial variation in day-to-day death reporting leads to more unstable *R*_*t*_ estimates over time. Calculating the correlation between mobility and *R*_*t*_ before and after the AKB period suggests a decoupling between transmission and mobility, whereby estimates of *R*_*t*_ during periods of equivalent levels of mobility during AKB are lower than estimates obtained before AKB.

### Understanding COVID-19 risk and subnational spread of SARS-CoV-2 across Java

**Fig. 3:**
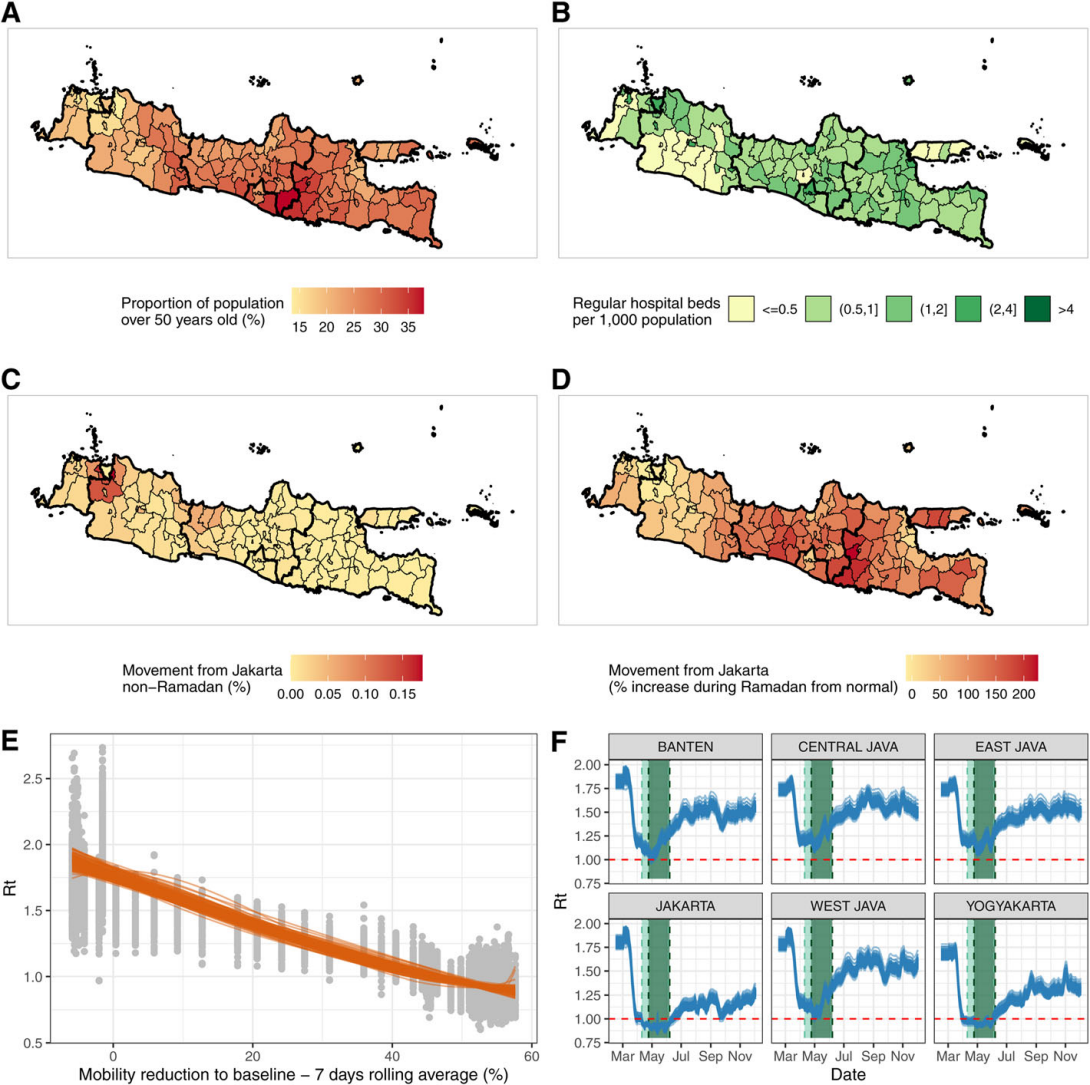
Key factors that are affecting the spread and severity of COVID-19 epidemic in Java, Indonesia. **a** Proportion of the population aged over 50 years old at the district level. **b** Number of regular hospital beds per one thousand population at the district level. **c** Proportion of Jakarta residents who spent their day in other districts in Java during a non-Ramadan period. **d** Increased proportion of people of Jakarta who spent their day in other districts in Java during Ramadan compared to the non-Ramadan period. **e** The relationship between the estimated *R*_*t*_ values based on C19P funerals data and average reduction in non-residential mobility in Jakarta using data only before the lifting of the first PSBB. Grey dots represent 100 samples of *R*_*t*_ values. Orange lines show the modelled smoothing spline relationship between 100 samples of *R*_*t*_ values and mobility reduction. **f** Extrapolations of *R*_*t*_ values in provinces in Java based upon Google Mobility trends for each province and the 100 sampled smoothing splines in Fig. 3e (orange lines). Light green shaded areas denote periods of PSBB whilst the dark green shaded areas represent the period of Ramadan domestic travel restrictions

Substantial variations exist across the island in terms of demography, healthcare capacity, and between-district mobility. The proportion of individuals over the age of 50 is typically higher (26%) in rural districts than urban ones (19%) (Fig. 3a). There are also substantial disparities in healthcare availability, ranging from the comparatively well-resourced Jakarta setting (2.22 hospital beds per thousand population) to the poorer, more rural setting of Tasikmalaya in West Java (0.18 beds per thousand population) (Fig. 3b). Patterns of between-district mobility outside of the window of the pandemic, estimated using mobile phone data over the period of 1 May 2011–30 April 2012, highlight the extent to which these settings are connected. Between-district connectivity is particularly high during the Ramadan period, with large-scale movements from densely populated Jakarta to other more rural regions with lower availability of healthcare (Fig. 3c, d). Applying our modelled relationship between mobility and *R*_*t*_ obtained from the Jakarta C19P funeral data (Fig. 3e) to trends in mobility data from the remaining provinces in Java suggests large reductions in transmission in all provinces coinciding with the first PSBB period (Fig. 3f). However, they also suggest that measures were sufficient to bring *R*_*t*_ below 1 for a sustained period only in Jakarta and Yogyakarta. Increases in mobility occurred either during early May (Banten, West Java, Central Java, and East Java) or alongside the establishment of the AKB in June (Jakarta and Yogyakarta), leading to corresponding increases in our estimates of *R*_*t*_ (Fig. 3f).

**Fig. 4:**
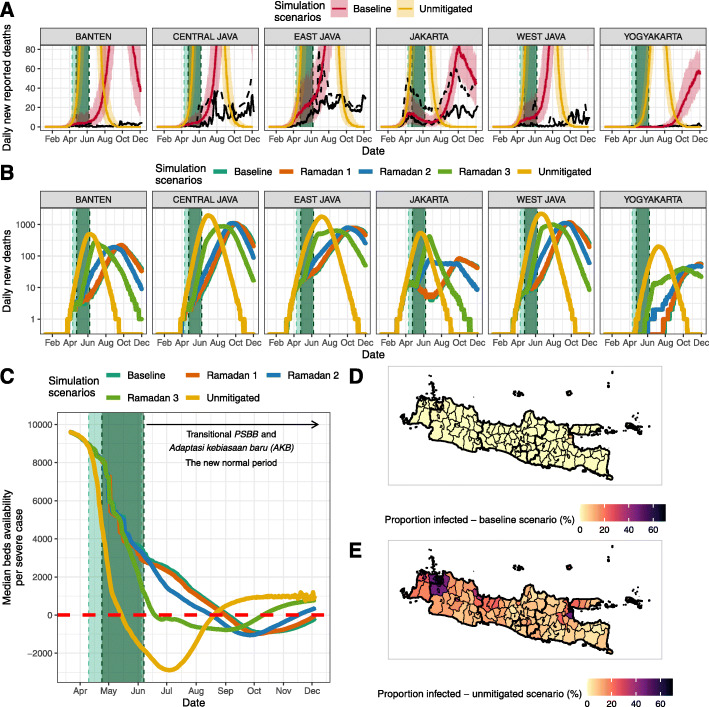
Metapopulation model simulation results. **a** Comparison of model simulations in the baseline scenario (red lines and their shaded 95% uncertainties ranges) and unmitigated scenario (yellow lines and their shaded 95% uncertainties ranges) and daily confirmed (solid black lines) and suspected (dashed black lines) deaths from COVID-19. **b** Model simulations in five different scenarios: (1) baseline scenario as shown in **a**, (2) Ramadan counter-factual 1 where it is assumed that there is no movement restrictions during the Ramadan period and *R*_*t*_ values are similar to the baseline scenario, (3) Ramadan counter-factual 2 where it is assumed that there is no movement restrictions during the Ramadan period and *R*_*t*_ values are 75% of each district’s *R*_0_ value, (4) Ramadan counter-factual 3 where it is assumed that there are no movement restrictions during the Ramadan period and *R*_*t*_ values are each district’s *R*_0_ value, and (5) unmitigated scenario where no interventions since the beginning of the epidemic are assumed. **c** Median hospital beds availability per severe COVID-19 case over time based on different simulation scenarios. **d** Proportion of people infected based on the actual scenario up to 31 May 2020 (before AKB/the ‘new normal’) at the district level. **e** Proportions of people infected based on the unmitigated scenario up to 31 May 2020 (before AKB) at the district level. Light green shaded areas denote periods of PSBB whilst the dark green shaded areas represent the period of Ramadan domestic travel restrictions

These estimates were integrated into our meta-population model (Fig. 4a). Estimates of deaths in the baseline scenario were consistent with observed qualitative patterns prior to the shift to the AKB phase of the epidemic in early June. The epicentre shifted over time from Jakarta to satellite towns and other provincial capitals and with Yogyakarta remaining least affected. Our baseline scenario’s median deaths fall within the range of cumulative confirmed and suspected deaths up to 31 May 2020 and the number of confirmed and suspected deaths between May 13 and May 31, 2020, in most provinces (Table 2). Total suspected deaths fell within the model’s uncertainty bounds for most provinces except Jakarta and Central Java (Table 2).

**Table 2:** Total number of estimated deaths based on model simulations of the baseline and unmitigated scenario

Province	Confirmed deaths May 13–31 (WHO Indonesia situation report 10 [4])	Suspected deaths May 13–31 (WHO Indonesia situation report 10 [4])	Baseline model scenario deaths May 13–31	Confirmed deaths up to May 31 [14, 31]	Suspected deaths up to May 31 (provincial data collated by KawalCOVID19 [32])	Baseline model scenario deaths up to May 31	Unmitigated counterfactual deaths up to May 31	Averted deaths up to May 31 (unmitigated–baseline)
Jakarta	74	447	158 (47–333)	520	2435	810 (292–1777)	16,356 (7896–21,593)	15,560 (7567–19,691)
West Java	46	351	197 (55–525)	135	653	525 (149–1368)	19,733 (5682–39,876)	19,151 (5516–38,400)
Central Java	4	269	88 (36–216)	66	666	203 (67–511)	6321 (2147–16,052)	6068 (2056–15,485)
Yogyakarta	0	1	2 (0–8)	9	29	6 (1–33)	401 (138–1097)	397 (132–1088)
East Java	241	458	437 (98–944)	395	1127	1091 (226–2646)	12,182 (3625–20,800)	10,997 (3277–18,102)
Banten	13	47	82 (20–224)	67	332	229 (66–711)	7302 (2180–14,732)	7079 (2111–14,141)
Java island total	378	1638	983 (360–1930)	1192	5242	2912 (1109–5851)	59,896 (26,787–112,795)	57,030 (24,843–105,378)

Values inside the brackets denote 95 percentile range of simulations. Suspected deaths are a combination of confirmed and probable COVID-19 deaths

The scenarios estimates are consistent with reductions in contact rates serving to reduce spread, reduce healthcare demand, and avert mortality prior to AKB phase: an estimated 57,000 (24,800–105,400, 95% UI) deaths averted when compared to an effectively unmitigated epidemic with *R*_*t*_ = 2 throughout this period (which we estimate would have resulted in 59,900 (26,800–112,800, 95% UI deaths). These numbers do not consider the effects of healthcare services becoming overwhelmed (as shown by the negative values of the median number of hospital beds available per COVID-19 case needing hospitalisation under the unmitigated epidemic scenario; Fig. 4c) on both direct and indirect mortality, an impact which would likely have been sizable given the wider spread to more rural settings with more scarce healthcare provision in our unmitigated scenario (Fig. 4d, e).

Our baseline scenario increasingly over-predicts deaths in most provinces during the AKB. This is in line with our results suggesting a decoupling of within-province mobility from virus transmissibility over that period.

### Estimating current COVID-19 burden, modelled future scenarios, and estimated vaccines impact in Java

Our projections generated 2 September 2020 [30] (Additional file 1: Figure S12 [15–26]) suggested that, whilst *R*_*t*_ was well below that observed at the beginning of the epidemic, this was driven primarily by the impact of control measures rather than the accumulation of population-level immunity. As a result, in the absence of additional control measures, death rates were likely to rise for the remainder of the year in all provinces, pushing all provinces beyond available hospital capacity. We found that reimplementation of PSBB could largely prevent capacity from being exceeded but would not prevent a subsequent wave if such control was not maintained.

**Fig. 5:**
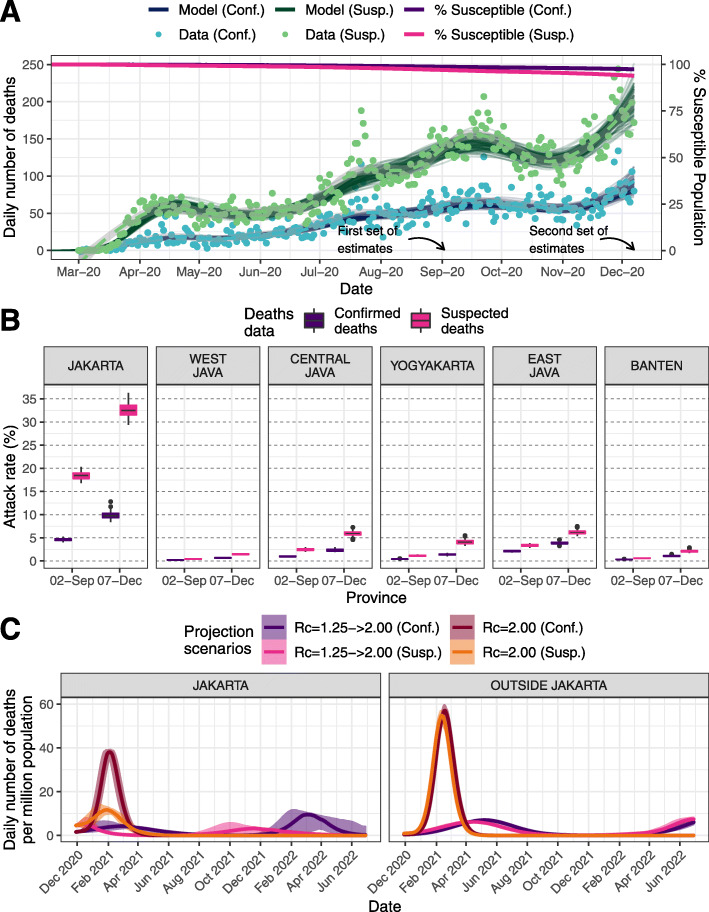
**a** Model fitting to confirmed and suspected (both confirmed and probable) COVID-19 related deaths and inferred population susceptibility in Java; green and blue dots show data on reported and suspected respectively (where suspected includes augmented estimate of probable deaths in provinces outside Jakarta), with associated median (lines) and 95% CrI (shaded areas) of model fits. **b** Estimated province-level attack rates (cumulative proportion infected) based on confirmed (purple) and suspected (pink) COVID-19 related deaths. **c** Projections of daily number of deaths due to COVID-19 based on four different transmission scenarios

Subsequently, between our two sets of simulations (2 September 2020 and 7 December 2020), both confirmed deaths and our inferred estimates of total suspected deaths increased from 5108 to 11,370 and 12,254 to 26,206, respectively, across Java. At the island level, the estimated attack rates on both time points increased from 1.21 to 2.57% and 2.95 to 6.03% based on confirmed deaths and assuming all suspected deaths as COVID-19 deaths, respectively (Fig. 5a). At the province level, estimates of attack rate and total burden from COVID-19 differ quite significantly, with Jakarta accumulating the highest attack rates in the region by 7 December 2020 (Fig. 5b; Additional file 1: Table S5 [15–26]). In all provinces and based on models fitted to either suspected or confirmed deaths, there were consistent increases of around 2–3 times on the province-level attack rate from 2 September to 7 December 2020. However, as seen at the island level, discrepancies between the estimated attack rates based on the model fitted to suspected deaths and confirmed deaths data were still observed at the province level, with the highest difference observed in Jakarta.

Projections of future scenarios from December 2020 (Fig. 5c; and Additional file 1: Figure S13 [15–26] for province-level breakdown), incorporating these changes in estimated attack rate and extrapolating current trends of *R*_*c*_, leads to the projected daily incidence of mortality across the island continuing to grow throughout the first half of the 2021 irrespective of whether reported or suspected mortality are more reflective of true direct COVID-19 mortality. In this scenario, with future *R*_*c*_ = 1.25, the epidemic would be projected to peak earliest in Jakarta, driven by the higher degree of population-level immunity implied by the higher cumulative attack rate to date. This peak’s timing was sensitive to the mortality metric the model is calibrated to, with projected peaks occurring early in 2021 for a current scenario based upon suspected deaths and towards the end of the first quarter of 2021 based upon reported deaths (Fig. 5c; and Additional file 1: Figure S13 [15–26]). Subsequently to these simulations, the incidence of COVID-19 deaths in Jakarta peaked in late January, prior to the rollout of the vaccine to the elderly in the province (Additional file 1: Figure S15 [15–26]), aligning more closely to our projections based upon these funeral data than those based upon confirmed deaths alone. Jakarta has also seen the largest and most steady subsequent decline of all provinces in Java (Additional file 1: Figure S16 [15–26]), supporting our model result that population-based immunity due to the mature stage of the epidemic is having the largest impact in this province. However, in all provinces, at no point in any of our current scenarios was there sufficient population-immunity to preclude a subsequent upsurge in deaths if transmission levels returned to those estimated at the beginning of the pandemic (*R*_*c*_ ≈ 2.00) prior to completion of an effective vaccination campaign.

**Fig. 6:**
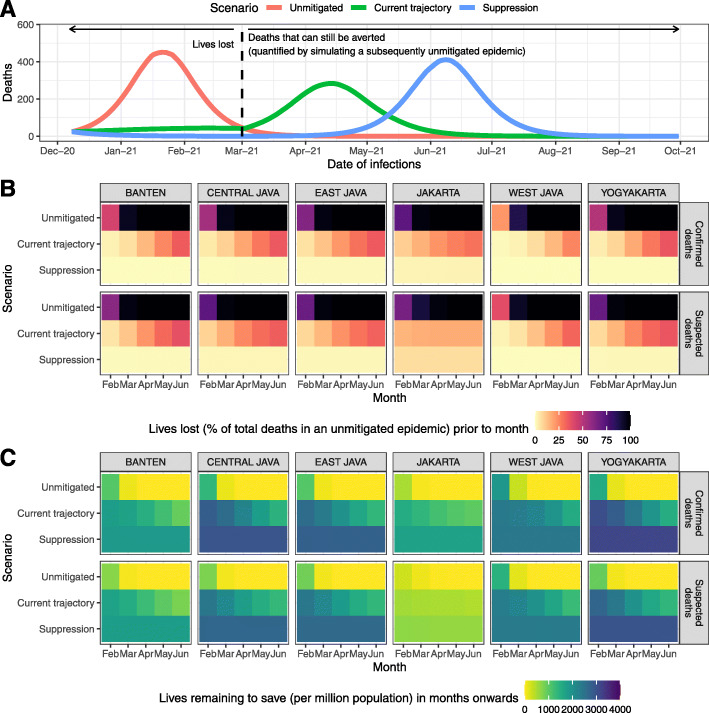
**a** An illustration of future scenario projections and how to define the number of lives lost and the number of deaths that can still be averted after a certain time point. The graph shows simulations based on a model fitted to confirmed COVID-19 deaths in Jakarta, which subsequently ‘returning to normal’ on 1 March 2021. **b** Projected percentage of lives lost (compared to total deaths from an unmitigated epidemic scenario) prior to the start of each month from February to June 2021, based on each simulation scenario and model fitted to confirmed or suspected deaths in each province in Java. **c** Projected number of lives remaining to be saved (or deaths that can still be averted) per million population after the start of each month from February to June 2021, based on each simulation scenario and model fitted to confirmed or suspected deaths in each province in Java

Figure 6a shows trajectories of the three different future scenarios summarized in terms of the proportion of lives lost before the beginning of a month (Fig. 6b) and the total remaining lives to be saved (deaths that can still be averted) after the start of the month (Fig. 6c). We estimate that reimposing suppression scenarios in areas where epidemics are on an upwards trajectory would significantly reduce lives lost during a period whilst the vaccine is rolled out. In some settings, such as Jakarta, assuming all suspected deaths were COVID-19 deaths, a combination of control measures currently in place and increasing levels of population immunity may combine to reduce transmission and burden to low-levels temporarily. At this point, the future incremental impact of suppression measures would likely be limited. However, in such scenarios, the need for ongoing NPIs as the vaccine is rolled out is highlighted by the high loss of life we estimate if such control measures are now lifted. This also highlights the substantial remaining incremental value of the vaccination campaign (Fig. 6c).

## Discussion

Our analysis uses C19P funeral data in Jakarta to highlight the considerable benefits of using syndromic measures of COVID-19 mortality to not only better measure the past but also to guide the future. Collecting such data is likely to yield high value for many countries where testing capacity has been severely strained in the face of the pandemic and for future pandemics of respiratory pathogens that require the development of new diagnostic capacity. C19P funerals and other measures of suspected mortality provide an alternative lens through which to understand COVID-19 burden and dynamics but do not allow precise measurement. Without confirmed diagnoses, the proportion of these individuals who were infected will always be unknown and liable to vary spatiotemporally, as will the extent to which measures of suspected deaths represent all deaths of individuals displaying COVID-19 symptoms. These data also support the substantial circulation of SARS-CoV-2 in Indonesia well before the first confirmed COVID-19 case [3] and the higher impact of the virus than suggested by confirmed deaths alone. Simultaneously, they also indicate an earlier decline in transmission during the early stages of the pandemic, coinciding with reductions in mobility and more sustained declines in transmissibility in response to NPIs than observed in confirmed deaths, a metric which is likely sensitive to limitations in testing. We also found these effects consistent with NPIs substantially attenuating spread across Java, including to older, more rural populations with lower access to healthcare.

Better quantifying impact in the past helps us to better understand likely scenarios in the future. In our first set of projections in September 2020 [30], we suggested that C19P funeral data could indicate up to a fourfold increase in cumulative exposure to the virus relative to confirmed deaths. However, even when assuming a higher burden of the disease in the population, immunity accumulated at the population level would not prevent the burden from increasing throughout the remainder of 2020. We also suggested that measures to suppress the virus could prevent such a scenario but would need to be sustained to prevent further upsurges. From early 2021, these projections appear to have been valid as transmission declined in Jakarta whilst PSBB was implemented between September 14 and October 11, 2020, but subsequently resurged once restrictions were lifted. At the beginning of 2021, Java’s epidemiological situation is substantially worse than in September, with record deaths reported week-on-week [33]. Subsequently, our projections, based upon C19P funeral data, of a likely decline in the epidemic in Jakarta independent from any vaccine impact has also come to fruition, again highlighting the utility of understanding the true impact of the disease in the population using syndromic measures of mortality.

Despite the qualitative validity of both our September and December 2020 projections, there are multiple limitations associated with these analyses that should be noted, particularly as our current estimates of attack rates in all provinces in Java have increased substantially. Firstly, it remains difficult to say what level of population-immunity is required to achieve herd immunity as individual immune responses to the virus are still not yet well understood (including strength and duration) [34, 35], and heterogeneity in population mixing beyond age-structure likely play important roles [36, 37]. Moreover, our estimates of counterfactual ‘return-to-normal’ scenarios rely upon an estimate of *R*_*c*_ = 2.00 from the early stage of the epidemic in Jakarta, a period in which data were particularly limited and where a degree of relevant behaviour change may have already occurred given increasing global concern around the pandemic. As this estimate is also below those estimated in the early stage of the epidemic from other settings [38], this estimate may represent a conservative measure of the basic reproduction number. These limitations around the inherent transmissibility and critical immunity threshold to control the virus need to be further considered in the light of recent concerns of new variants of concern across the globe which appear more transmissible [39–41]. There have also been observed resurgences in populations where attack rates have likely passed many estimates of the herd immunity threshold [39, 42].

The initiation of the vaccination campaign in the middle of January [8] provides hopes for more sustainable control of the virus. However, challenges in access and distribution [43] and uncertainty in vaccine efficacy [43] could hamper the life-saving impact of the vaccination programme. On top of that, possible introductions of variants of concern (VOC) able to escape immune response [44], exhibit increased transmissibility [45, 46], or cause more severe disease [47] could also bring additional challenges in controlling the spread of the virus. Those changes in virus characteristics could lower the real-life vaccine effectiveness, increase the threshold to reach ‘herd immunity’, and threaten the healthcare capacity once again if infections from those VOC were able to dominate and take off. To date, Indonesia had reported several VOC (i.e., B.1.1.7 [48], B.1.525 [49], and a variant with E484K mutation [50]) via a ramp-up of genomic surveillance capacity at the turn of the year [51]. Whilst the sporadic nature of the genomic samples makes it difficult to determine whether local transmission had been established or not [51], such VOCs further underline the need to maintain control whilst the vaccination campaign is ongoing.

Despite the unprecedented speed of global vaccine development, our study indicates that in the absence of NPIs implemented over the previous year, this campaign would have been too late to prevent most deaths that currently remain avertable. It also highlights the ongoing value and need to maintain current control measures during the coming months as the vaccine is rolled out. Given low estimated attack rates and current increasing trends in transmission across much of the island, our results suggest that further measures aimed towards suppression of the disease over the next few months would substantially increase the proportion of the population who receive the vaccine prior to being exposed to infection, leading to a likely substantial incremental impact of the vaccination campaign. However, we are not able to capture the socio-economic costs of such approaches, which would need to be factored into balanced decision-making.

The case for maintaining or increasing control measures is likely more intuitive to grasp in circumstances where the incidence of cases and deaths continues to rise. However, our projections for Jakarta, particularly those incorporating suspected deaths, suggest that population-level immunity is contributing largely to the decline in observed deaths. This effect may have consequences for the perceptions of both the vaccine’s relative impact, with deaths declining at a faster rate in Jakarta relative to other provinces as the vaccine is being rolled out, as well as the ongoing need for NPIs and/or high vaccine uptake. In such circumstances, our counterfactual of a ‘return-to-normal’, which produces major upsurges in cases and deaths in every province regardless of mortality metric, provides a valuable reminder that the epidemic, and the need to control it, is far from over in any region of Java.

## Conclusions

This study gives evidence of the value of syndrome-based mortality as a metric, which is less dependent upon testing capacity with which to estimate transmission trends and evaluate intervention impact. NPIs implemented in Java earlier in the pandemic have substantially slowed the course of the epidemic with movement restrictions during Ramadan preventing spread to more vulnerable rural populations. Further relaxation of measures would lead to more rapidly progressing epidemics, depleting the eventual incremental effectiveness of the vaccine. Maintaining adherence to control measures in Jakarta may be particularly challenging if the epidemic enters a decline phase but will remain necessary to prevent a subsequent large wave. Elsewhere, higher levels of control with NPIs are likely to yield high synergistic vaccine impact. Enduring vigilance is vital whilst the vaccination campaign is rolled out, especially in light of the emergence of VOC across the globe.

## Supplementary Information

**Additional file 1: Figure S1-S16** and **Table S1-S5** of ‘Using syndromic measures of mortality to capture the dynamics of COVID-19 in Java, Indonesia in the context of vaccination rollout’. This additional file comprises of all supplementary figures and tables accompanying the main text.

**Additional file 2.** Supplementary methods section of ‘Using syndromic measures of mortality to capture the dynamics of COVID-19 in Java, Indonesia in the context of vaccination rollout’. This additional file comprises of all supplementary methods accompanying methods described in the main text.

## Data Availability

Full details of the data used in this article and the methods to analyse them are provided in the Additional file 2. The datasets supporting the conclusions of this article are available in: https://github.com/andradjaafara/covid19_indonesia_data.

## Abbreviations

AKB: *Adaptasi Kebiasaan Baru* (in Indonesian); the ‘new normal’ period
C19P: COVID-19 protocols
NPIs: Non-pharmaceutical interventions
PSBB: *Pembatasan Sosial Berskala Besar* (in Indonesian); large-scale social restrictions (English translation)
SEIR: Susceptible-Exposed-Infected-Recovered
WHO: World Health Organization
