# Supplementary material for: Using syndromic measures of mortality to capture the dynamics of COVID-19 in Java, Indonesia, in the context of vaccination rollout
Source: BMC Med. 2021 Jun 18;19:146. doi: 10.1186/s12916-021-02016-2 (PMC8212796; doi:10.1186/s12916-021-02016-2)
Supplement: Supplementary file 1 — Additional file 1: Figure S1-S16 and Table S1-S5 of ‘Using syndromic measures of mortality to capture the dynamics of COVID-19 in Java, Indonesia in the context of vaccination rollout’. This additional file comprises of all supplementary figures and tables accompanying the main text. [file 12916_2021_2016_MOESM1_ESM.docx]

**Additional File 1**


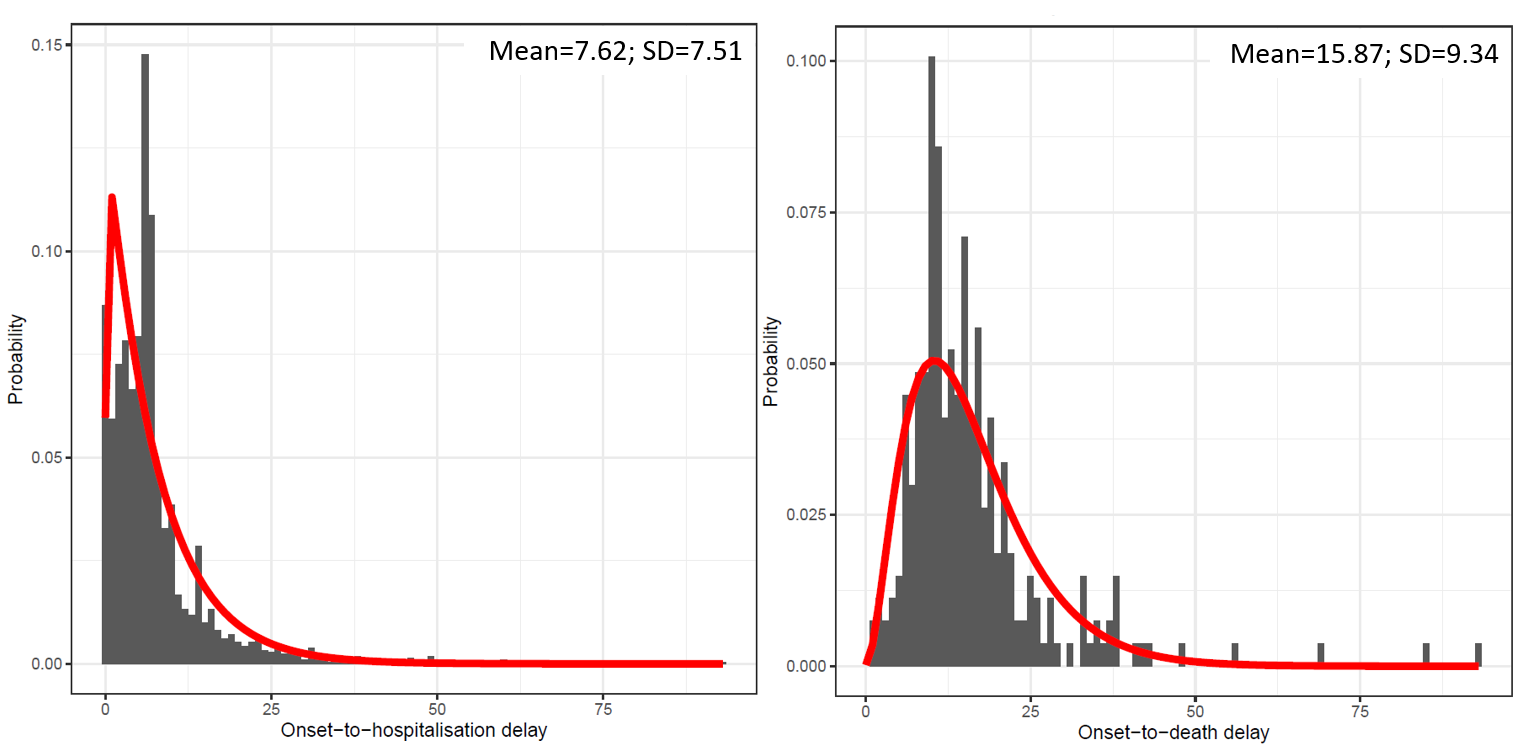


**Figure S1. Discretised Gamma distribution fittings to onset-to-hospitalisation delay data (left) and onset-to-death delay data (right).** Details on the model fitting methods on Additional File 2: S2 Reconstruction of frequency of onset.


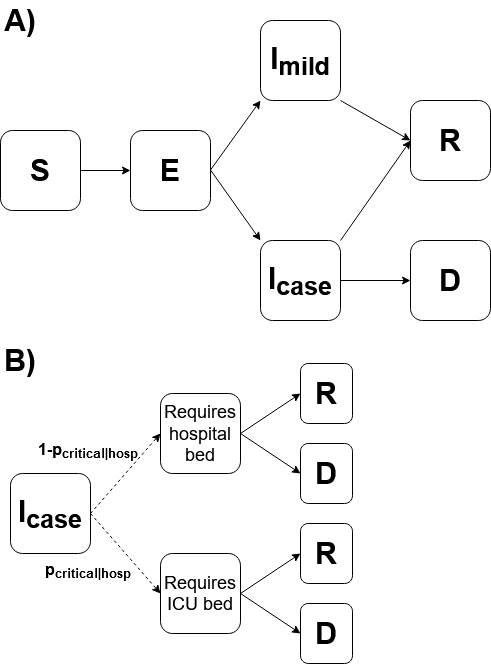


**Figure S2. SEIR model structure. A)** The SEIR structure for each patch in the metapopulation model. Susceptible individuals ($\boldsymbol{S}$), if infected, progress to the exposed compartment ($\boldsymbol{E}$), having their latent period of infections. Then, those individuals will either develop mild symptoms ($\boldsymbol{I}_{\boldsymbol{mild}}$) or severe symptoms requiring hospitalisations ($I_{case}$). Those who developed severe symptoms may have two possible outcomes: recovery ($R$) or death ($D$); **B)** The pathway of infections requiring hospitalisations ($I_{case}$). Each infection with severe symptoms may only need a standard hospital bed or may develop worse conditions that require critical care (ICU bed). Each of those cases treated in both critical and non-critical care may recover or die based on specific probabilities. Dashed lines denote probabilistic pathways, not rates of transition.


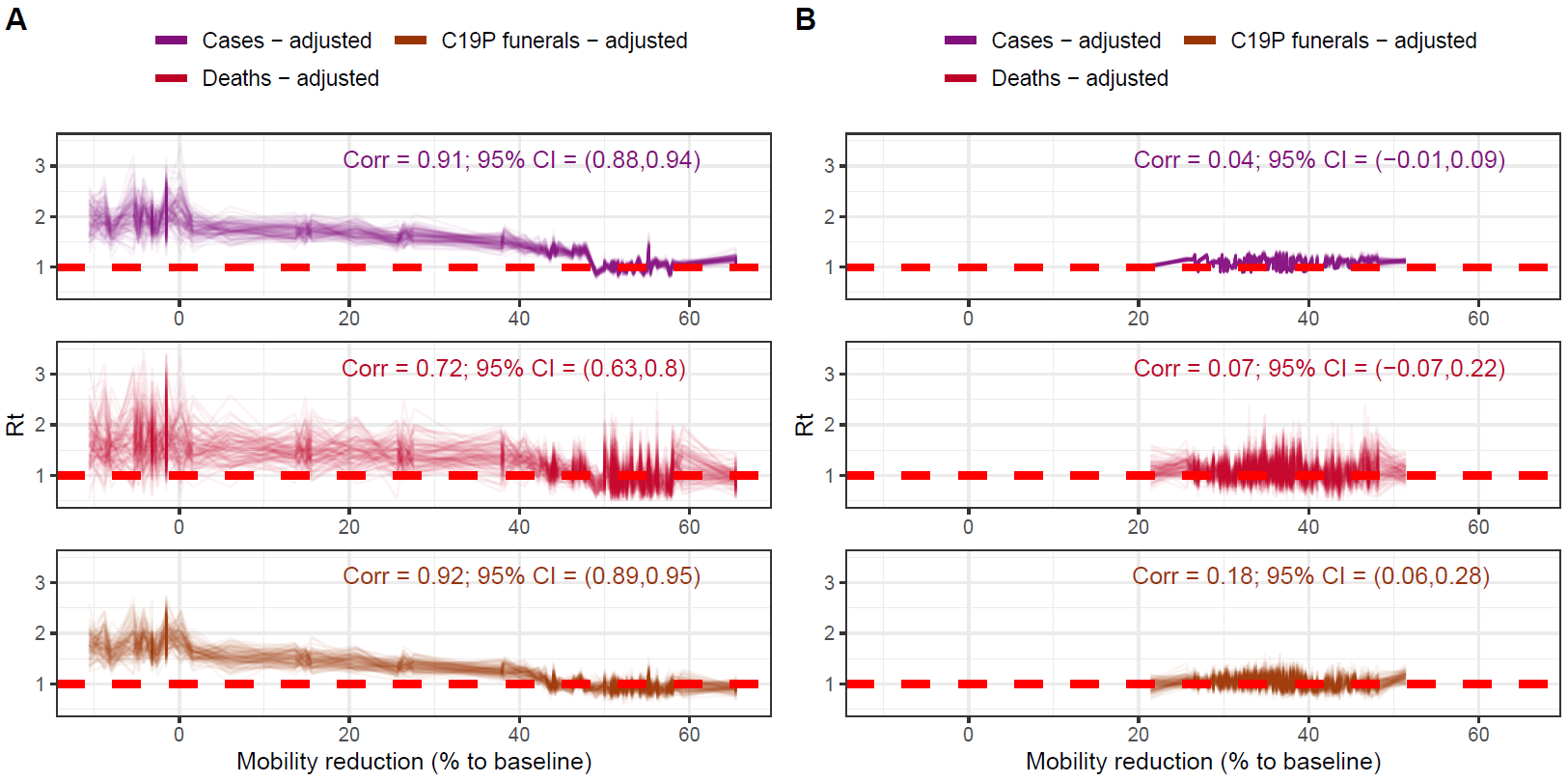


**Figure S3**. **Correlations between estimated** $\boldsymbol{R}_{\boldsymbol{t,funerals}}$ **in Jakarta and mobility changes based on** $\boldsymbol{R}_{\boldsymbol{t,funerals}}$ **estimates.** Before AKB (the ‘new normal’) **(A)** and after AKB **(B)**. Decorrelations between two metrics were observed after AKB (or the lifting of the first PSBB).


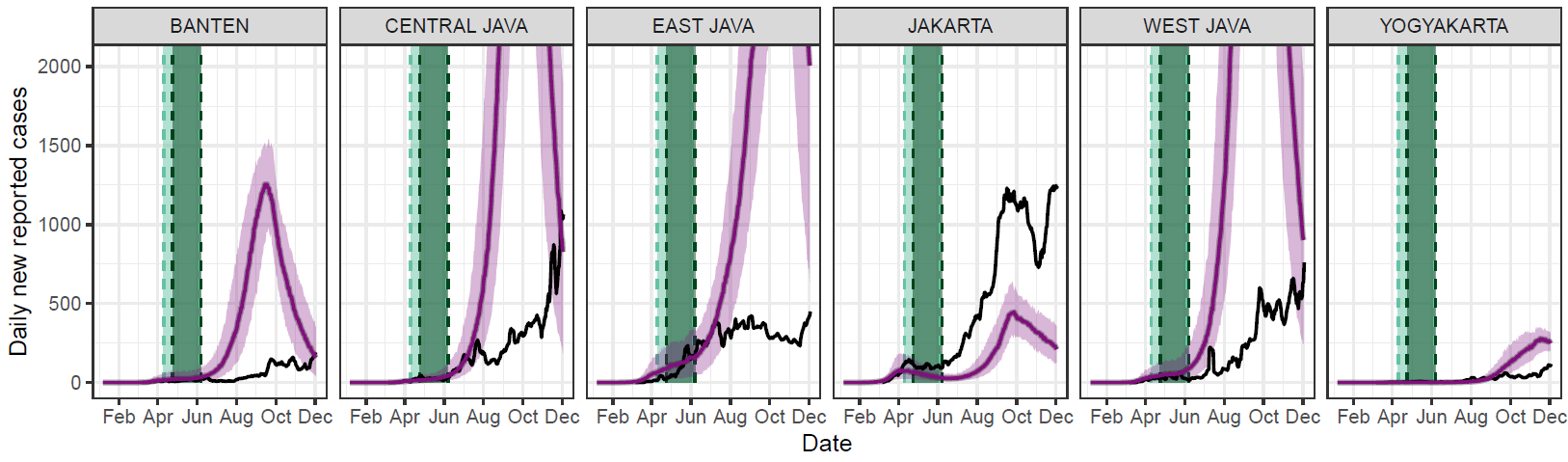
**Figure S4.** **Comparison of model simulations and observed daily new reported cases from COVID-19.** Coloured lines and their shaded areas denote model simulation outputs with their respective uncertainties (95% level) while black lines denote observed data. Detailed simulation scenarios on Additional file 1: Table S3 and Table 1 of the main text. Light green shaded areas denote periods of PSBB while the dark green shaded areas represent the period of Ramadan domestic travel restrictions.


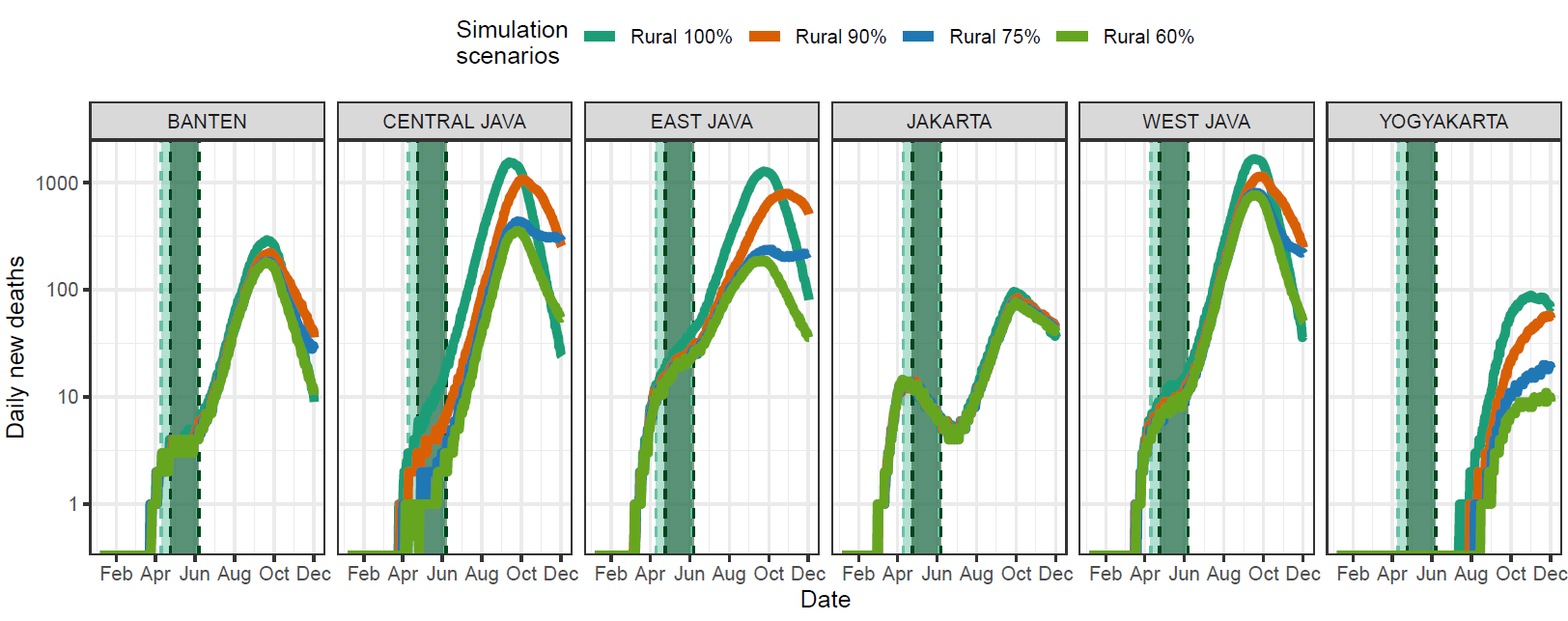
**Figure S5. Simulated daily new deaths comparing rural transmission scenarios at the province-level.** Detailed simulation scenarios on Additional file 1: Table S3. Light green shaded areas denote periods of PSBB while the dark green shaded areas represent the period of Ramadan domestic travel restrictions.


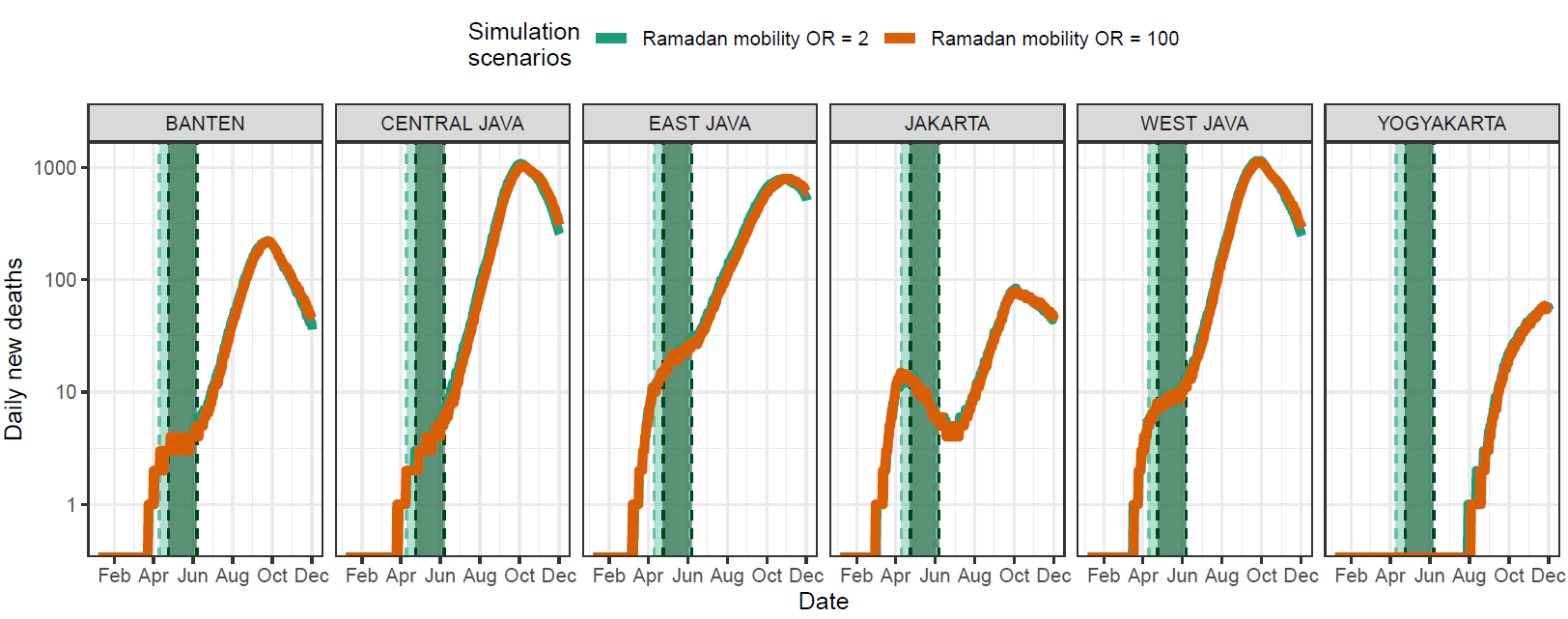
**Figure S6.** **Sensitivity analysis of assumed impact of between-district movement restrictions at the province-level.** Detailed simulation scenarios on Additional file 1: Table S3. Light green shaded areas denote periods of PSBB while the dark green shaded areas represent the period of Ramadan domestic travel restrictions.


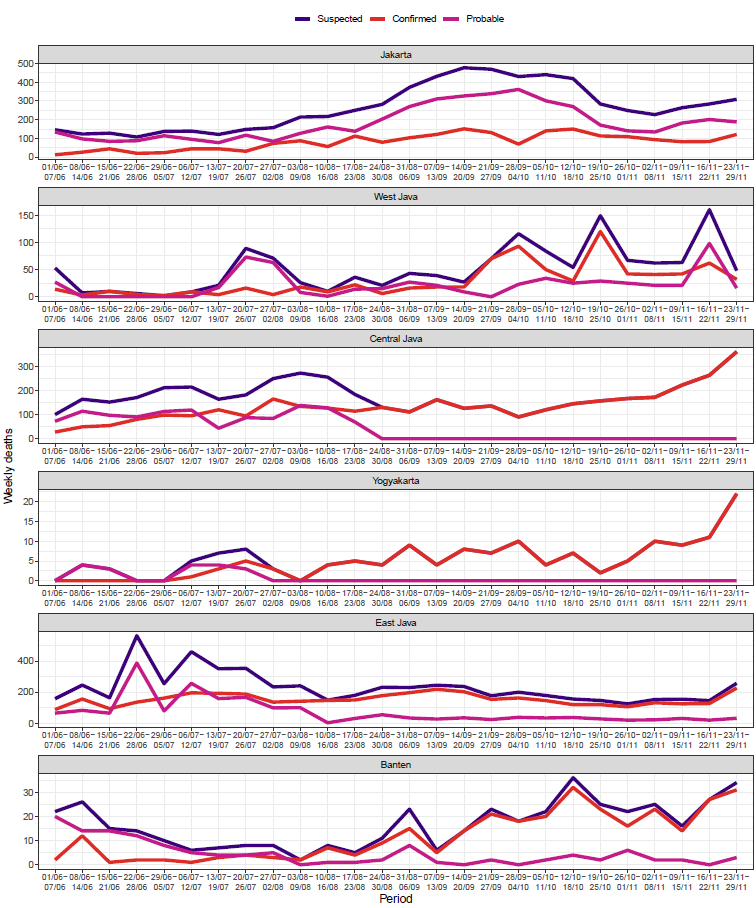


Figure S7. Weekly aggregated reported/confirmed and probable deaths in all six provinces of Java. Data collated from WHO COVID-19 Indonesia situation reports 13-36 [1].


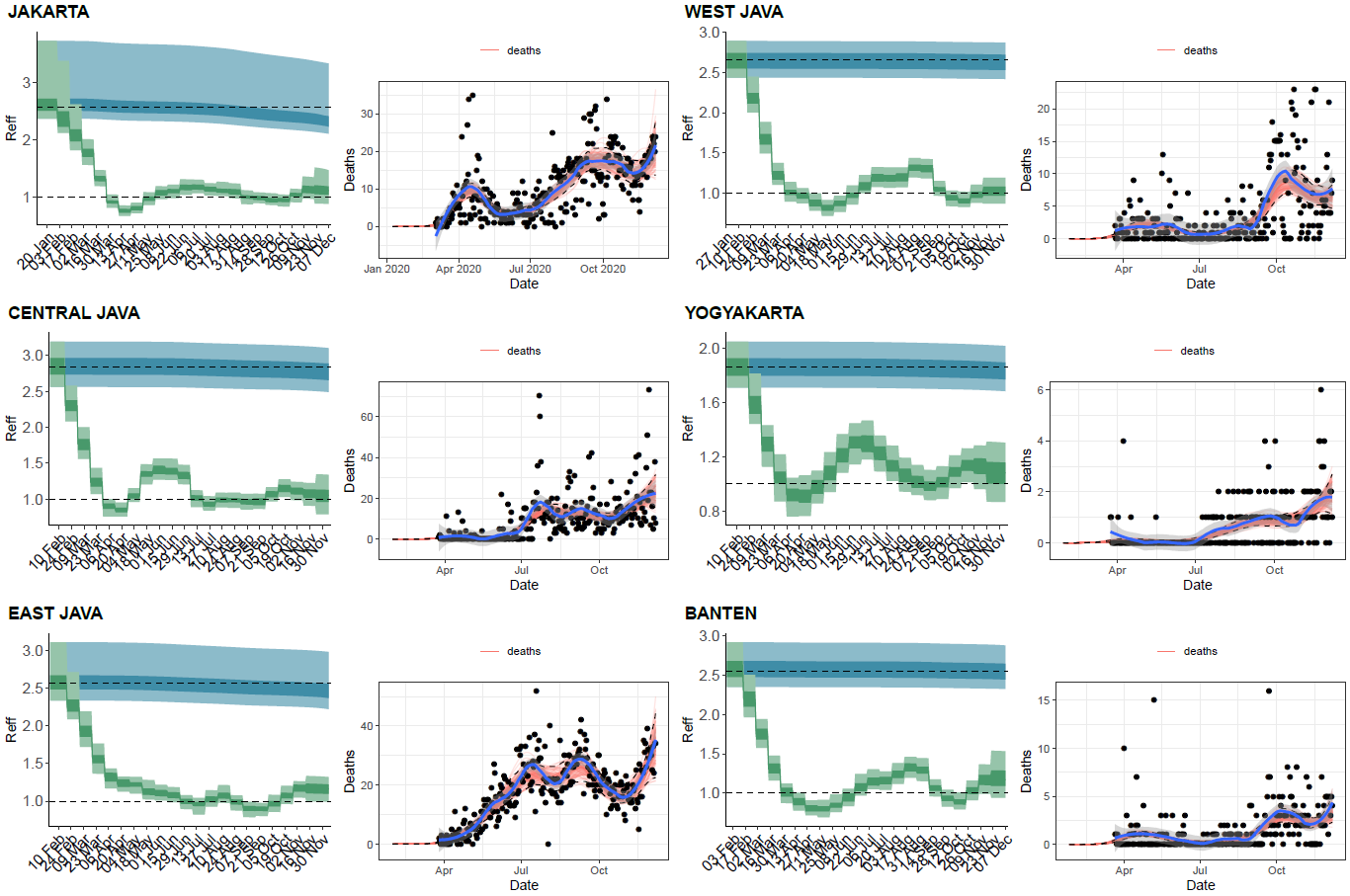


Figure S8. Model fits to reported deaths data and estimated $\boldsymbol{R}_{\boldsymbol{c}}$ values. Details on the model fitting methods on Additional File 2: S6 Model fitting to confirmed and suspected COVID-19 deaths and future projection scenarios in all provinces in Java.


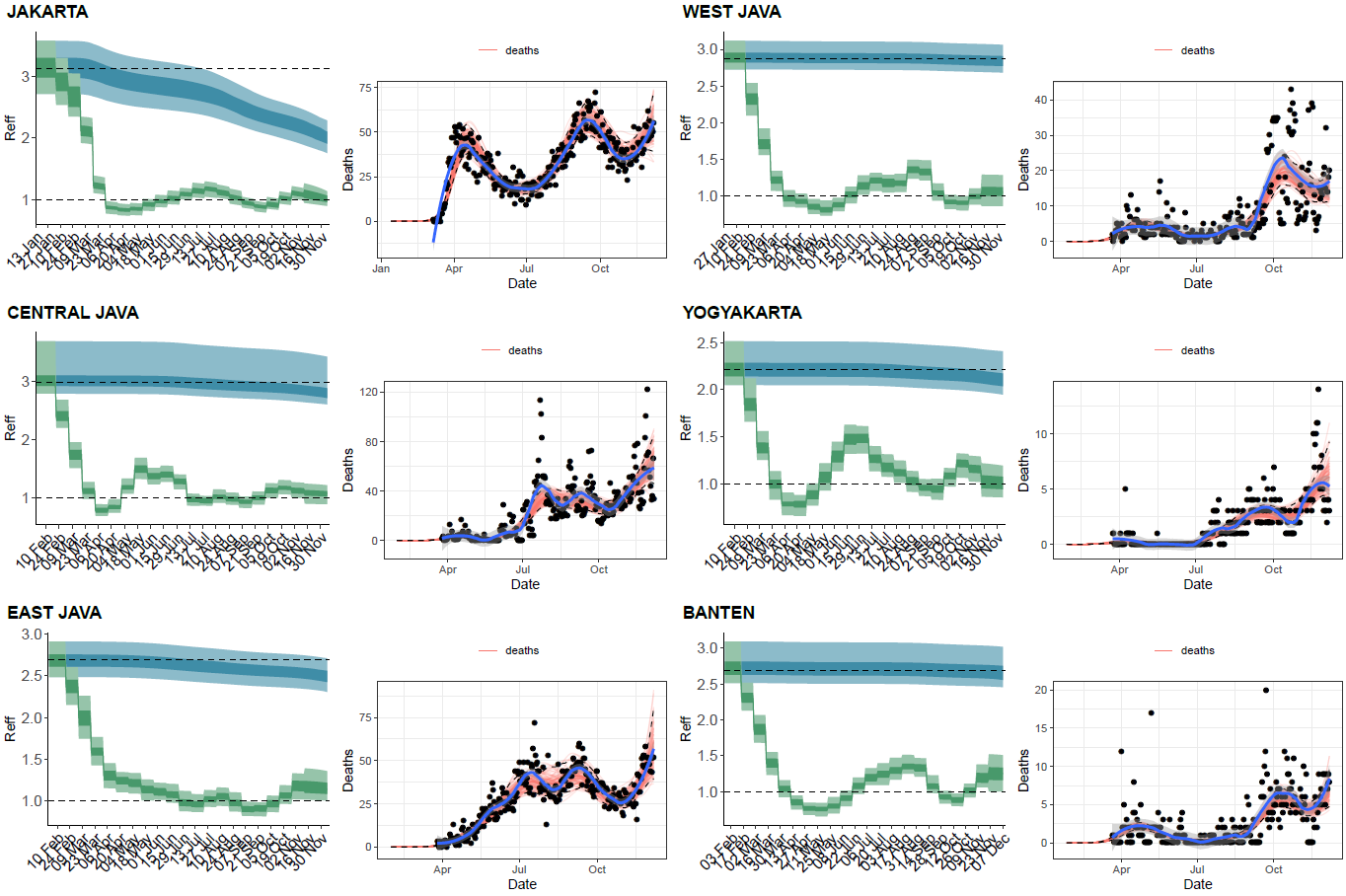


Figure S9. Model fits to suspected deaths data and estimated $\boldsymbol{R}_{\boldsymbol{c}}$ values. Details on the model fitting methods on Additional File 2: S6 Model fitting to confirmed and suspected COVID-19 deaths and future projection scenarios in all provinces in Java.


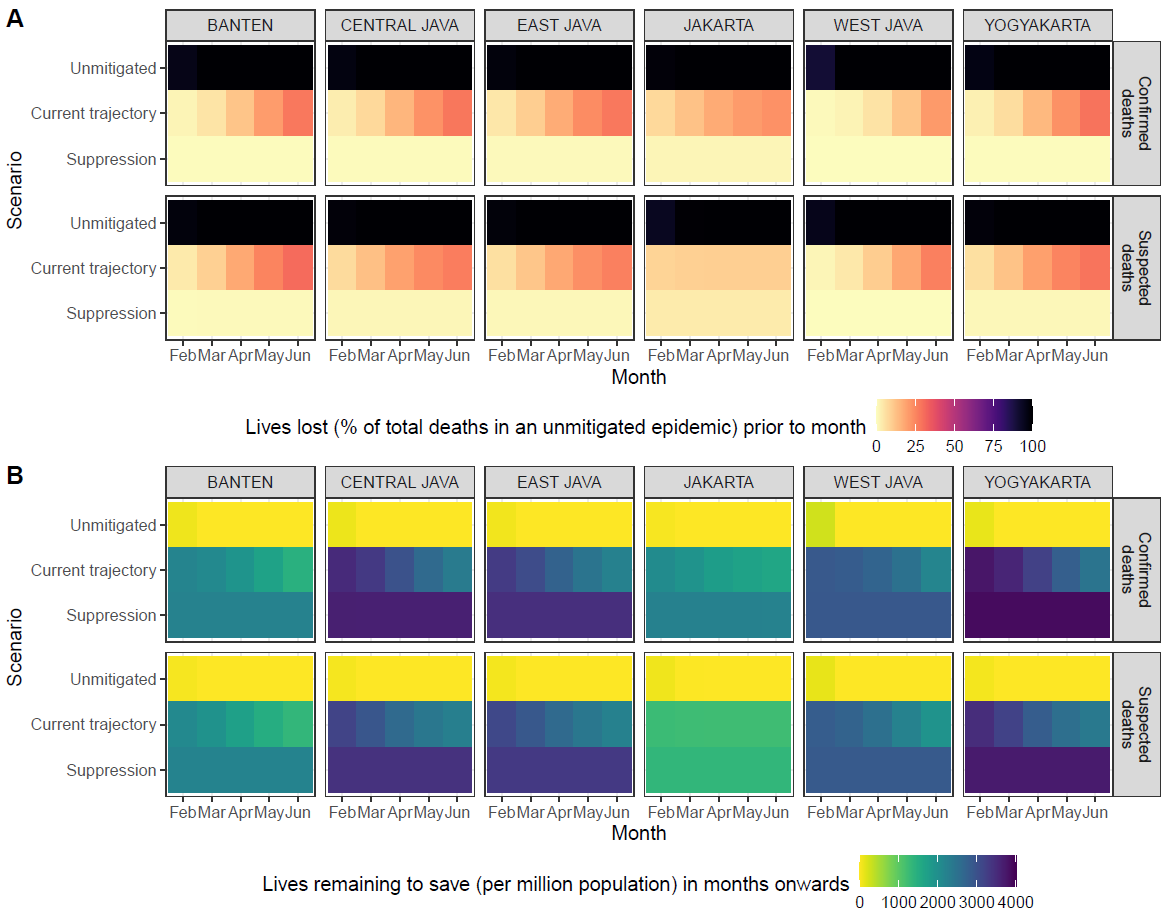


Figure S10. Additional analysis for projections of the number of lives lost and remaining to save (compared to Figure 6 in the main text), based on the assumptions of a higher reproduction number when ‘returning to normal’ and the epidemic is unmitigated ($\boldsymbol{R}_{\boldsymbol{c}}\boldsymbol{=3.00}$). A) Projected percentage of lives lost (compared to total deaths from an unmitigated epidemic scenario) prior to the start of each month from February to June 2021, based on each simulation scenario and model fitted to confirmed or suspected deaths in each province in Java; B) Projected number of lives remaining to be saved (or deaths that can still be averted) per million population after the start of each month from February to June 2021, based on each simulation scenario and model fitted to confirmed or suspected deaths in each province in Java.


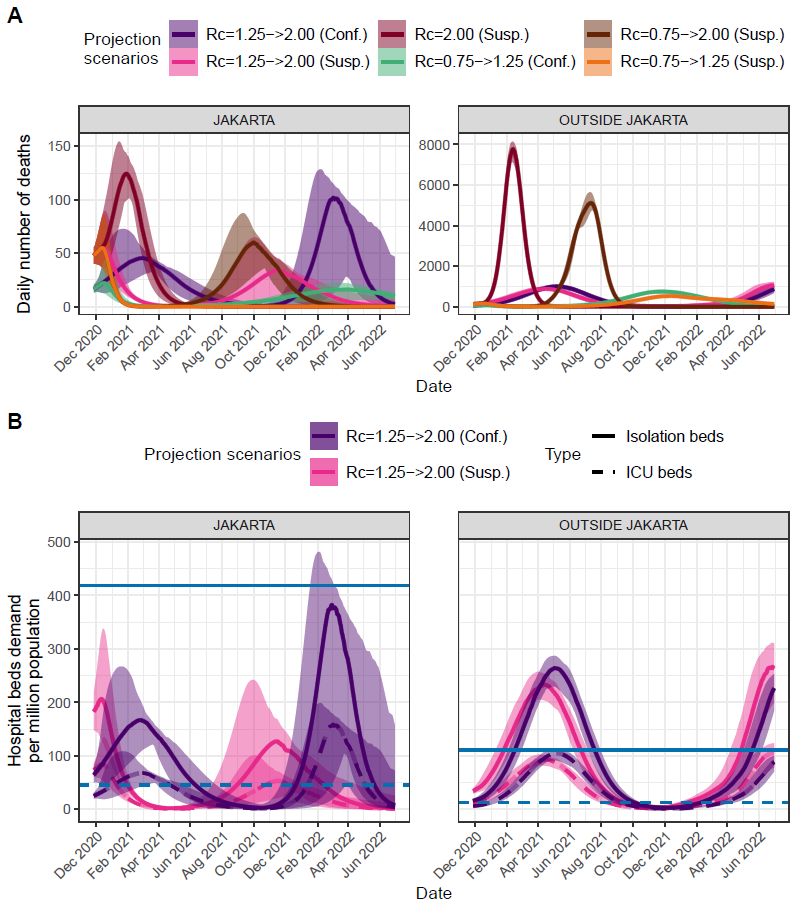


Figure S11. Future projections of deaths and healthcare demands based on different transmission scenarios. A) Projections of daily number of deaths due to COVID-19; B) Healthcare demand projections in the form of isolation beds and ICU beds demands assuming the current level of transmission to continue in the future (with easing of control measures after the transmission reached a low-level following the first peak in the graph). Projections are coloured according to whether they are based upon confirmed or suspected deaths to date and by projected $\boldsymbol{R}_{\boldsymbol{c}}$ (with $\boldsymbol{R}_{\boldsymbol{c}}\boldsymbol{=x\to y}$ representing $\boldsymbol{R}_{\boldsymbol{c}}\boldsymbol{=x}$ for immediate future and $\boldsymbol{R}_{\boldsymbol{c}}\boldsymbol{=y}$, the level it returns to when burden falls below 7 deaths per week). Healthcare capacities are based on the current numbers of dedicated COVID-19 isolation beds and ICUs [2], not reflecting the total number of beds and ICUs in each province.


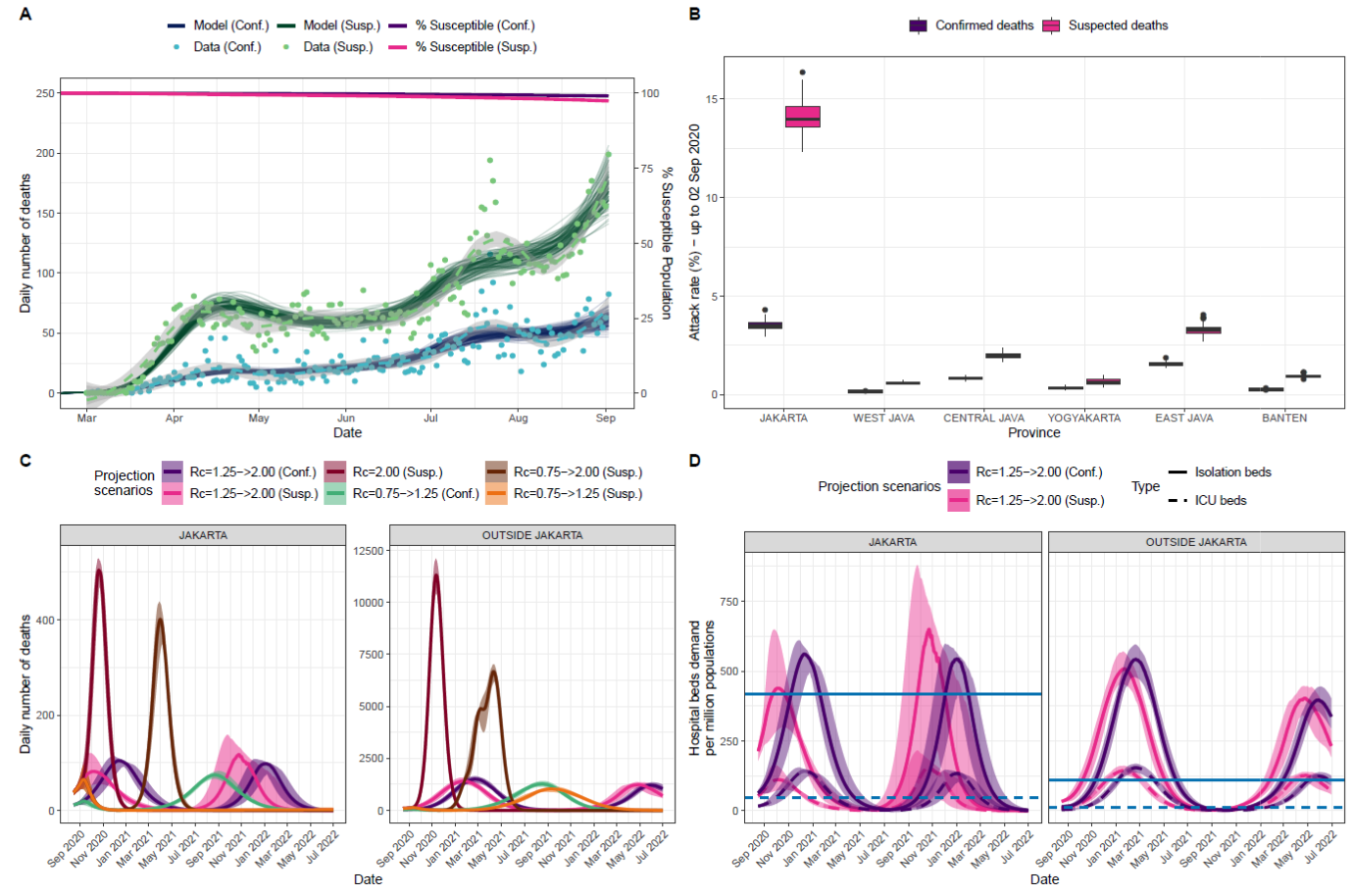


**Figure S12**. **The figure was taken from Djaafara et. al.** [3] **(Figure 5).** A) Model fitting to confirmed and suspected (both confirmed and probable) COVID-19 related deaths and inferred population susceptibility in Java; Green and blue dots show data on reported and suspected respectively (where suspected includes augmented estimate of probably deaths in provinces outside Jakarta pre-May 13th), with associated median (lines) and 95% CrI (shaded areas) of model fits. B) Estimated province-level attack rates (cumulative proportion infected) based on confirmed (purple) and suspected COVID-19 related deaths. C) Projections of daily number of deaths due to COVID-19 based on four different transmissibility scenarios. D) Healthcare demand projections in the form of isolation beds and ICU beds demands assuming the current level of transmission to continue in the future (with easing of control measures after the transmission reached a low-level following the first peak in the graph). Projections are coloured according to whether they are based upon confirmed or suspected deaths to date and by projected $R_{c}$ (with $R_{c}=x->y$ representing $R_{c}=x$ for immediate future and $R_{c}=y$, the level it returns to when burden falls below 7 deaths per week). Healthcare capacities are based on the current numbers of dedicated COVID-19 isolation beds and ICUs,[2] not reflecting the total number of beds and ICUs in each province.


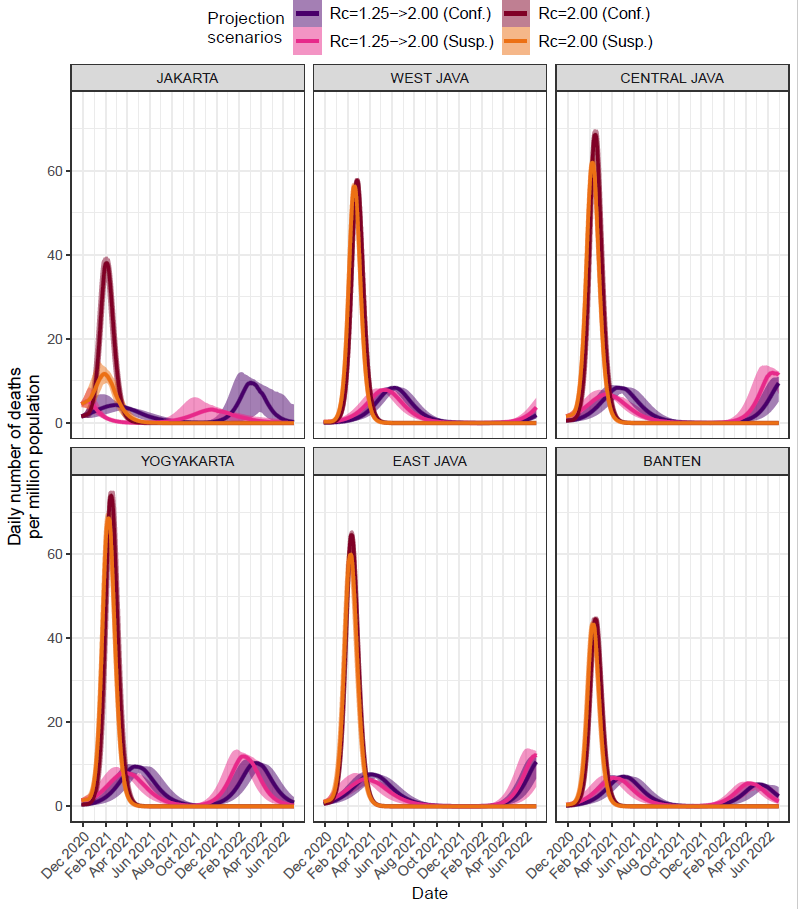
**Figure S13**. **Projections of daily number of deaths due to COVID-19 based on four different transmission scenarios as shown in Figure 5C in the main text but showing all provinces in Java.** $R_{c}=1.25$ represents ‘current trajectory’ scenario.


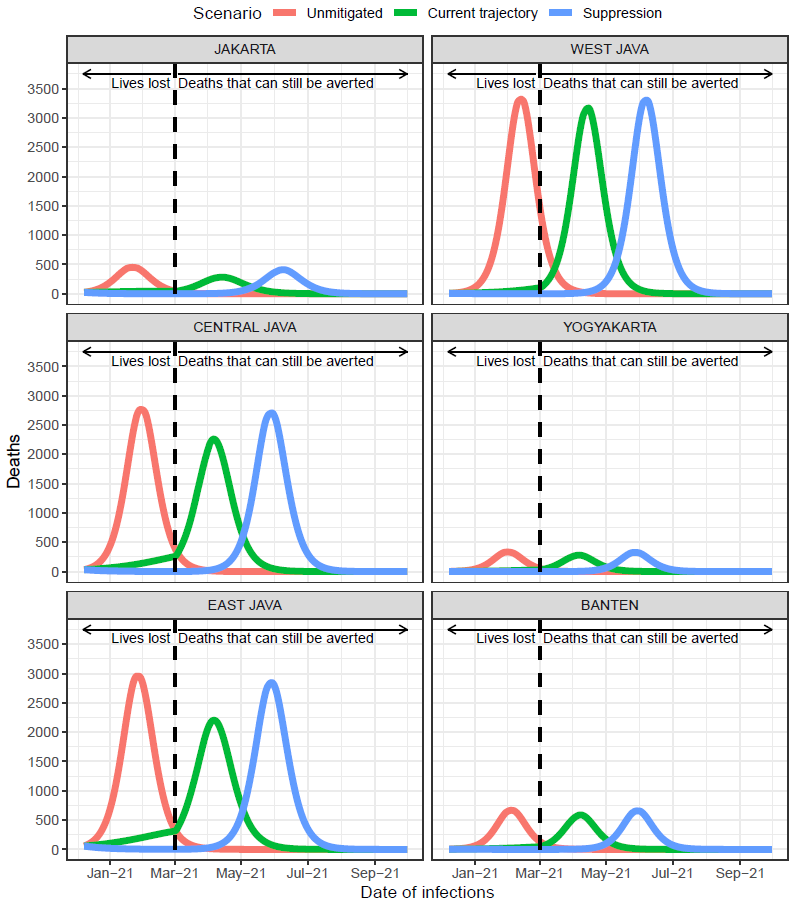
Figure S14. Illustrations of future scenario projections based on models fitted to confirmed COVID-19 deaths in each province in Java which subsequently ‘returning to normal’ on 1^st^ March 2021. The Jakarta trajectories are similar to what are shown in Figure 6A of the main text.

**
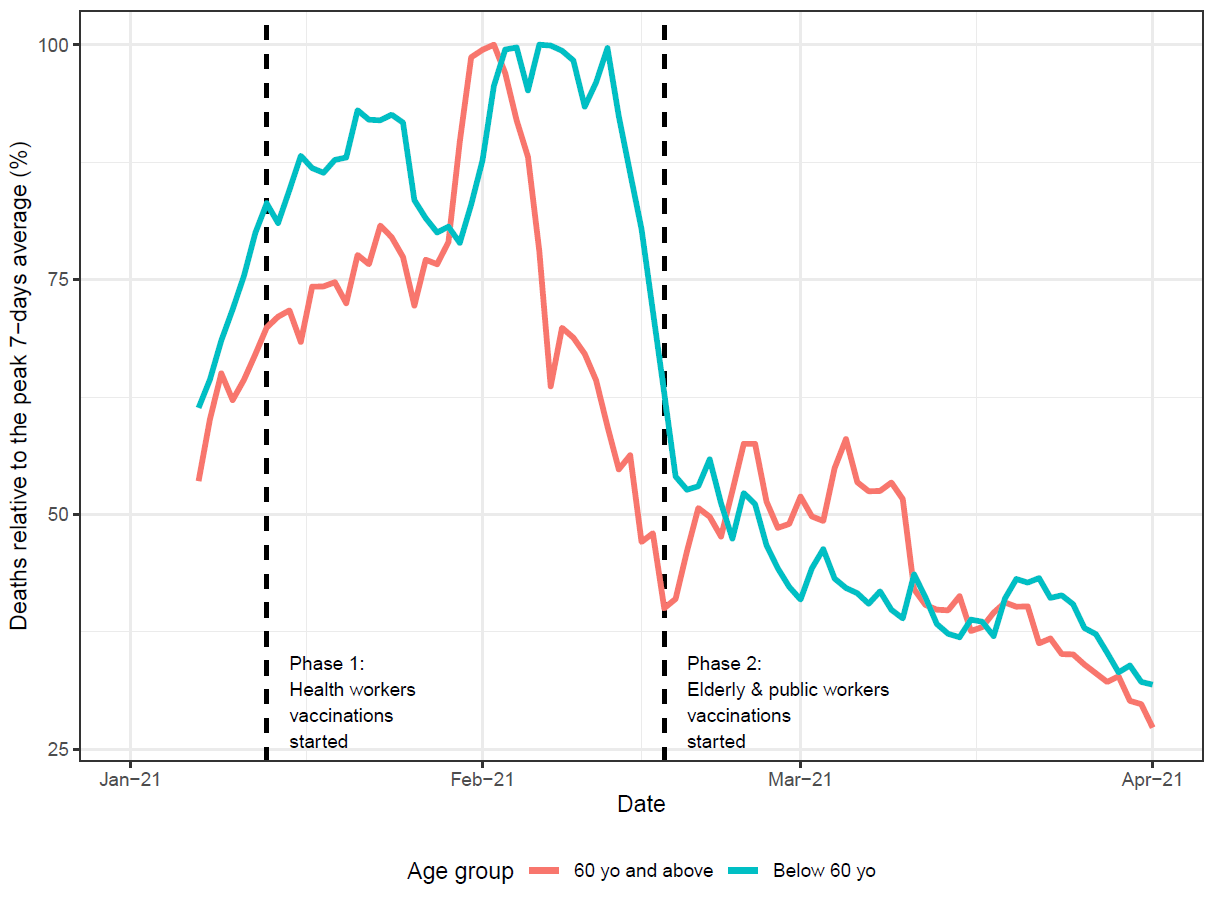
**

**Figure S15.** **Daily COVID-19 deaths as a relative proportion (%) to the peak of the epidemic in early 2021 in Jakarta for eldery (aged 60 years old and above) and non-elderly (aged below 60 years old).** 7-days moving average of the daily reported COVID-19 deaths were used for the calculations. Vaccinations for health care workers started on 13^th^ January 2021. Vaccinations for eldery and public workers started on 17^th^ February 2021. Data were obtained from the Jakarta Provincial Department of Health.


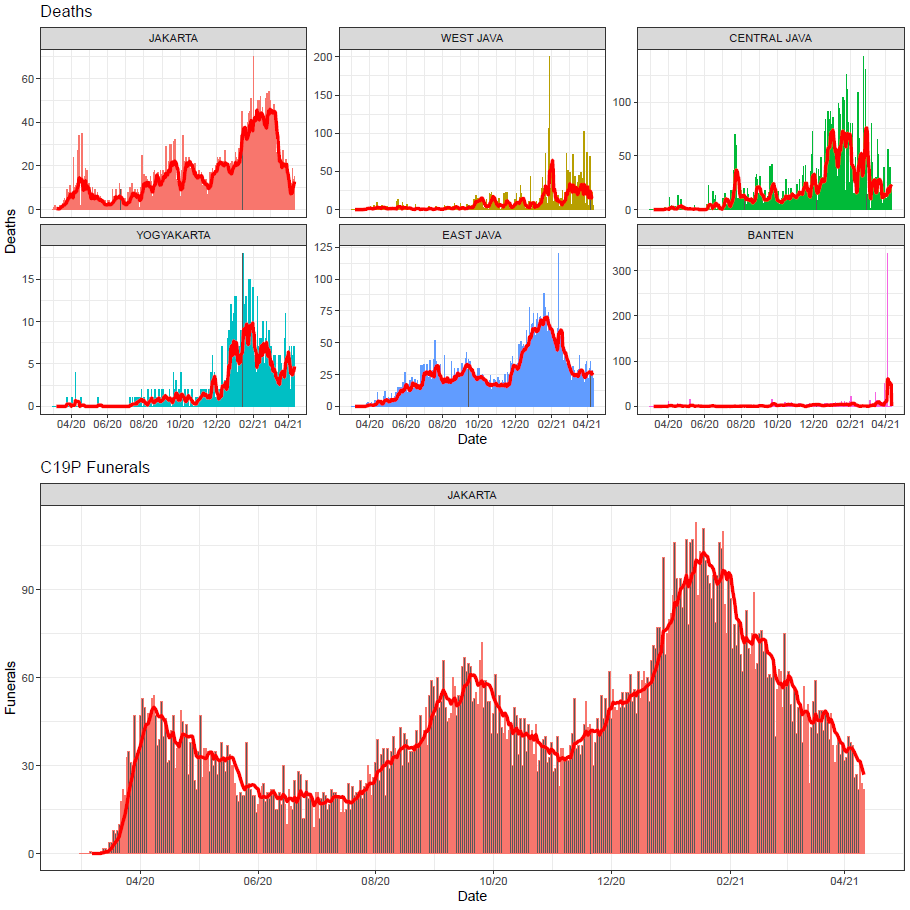


Figure S16. Daily reported number of COVID-19 deaths in all provinces in Jakarta up to 11 April 2021 and daily number of C19P funerals in Jakarta up to 11 April 2021. Red lines are the 7-days moving averages of the reported number of COVID-19 deaths/C19P funerals. Jakarta data were obtained from Jakarta COVID-19 data monitoring website [4]. Other provinces data were obtained from KawalCOVI19 online spreadsheet [5].

**Table S1. List of districts, districts’ indexes, and probability of disease severity and outcomes for the metapopulation model.**

| **Index no.** | **District** | **Province** | $\boldsymbol{p}_{\boldsymbol{hosp,i}}$ | $\boldsymbol{p}_{\begin{aligned} \boldsymbol{critical\vert} \\ \boldsymbol{hosp,i} \end{aligned}}$ | $\boldsymbol{p}_{\begin{aligned} \boldsymbol{death\vert} \\ \boldsymbol{non critical,i} \end{aligned}}$ |
| --- | --- | --- | --- | --- | --- |
| 1 | Jakarta | Jakarta | 0.029 | 0.185 | 0.054 |
| 2 | Outside Java | Outside Java | 0.027 | 0.195 | 0.058 |
| 3 | Bogor | West Java | 0.026 | 0.190 | 0.056 |
| 4 | Sukabumi | West Java | 0.031 | 0.216 | 0.065 |
| 5 | Cianjur | West Java | 0.031 | 0.212 | 0.064 |
| 6 | Bandung | West Java | 0.028 | 0.201 | 0.060 |
| 7 | Garut | West Java | 0.030 | 0.219 | 0.067 |
| 8 | Tasikmalaya | West Java | 0.034 | 0.228 | 0.070 |
| 9 | Ciamis | West Java | 0.039 | 0.234 | 0.072 |
| 10 | Kuningan | West Java | 0.036 | 0.233 | 0.072 |
| 11 | Cirebon | West Java | 0.030 | 0.209 | 0.063 |
| 12 | Majalengka | West Java | 0.036 | 0.229 | 0.070 |
| 13 | Sumedang | West Java | 0.036 | 0.234 | 0.073 |
| 14 | Indramayu | West Java | 0.033 | 0.210 | 0.063 |
| 15 | Subang | West Java | 0.035 | 0.224 | 0.069 |
| 16 | Purwakarta | West Java | 0.029 | 0.206 | 0.061 |
| 17 | Karawang | West Java | 0.030 | 0.200 | 0.059 |
| 18 | Bekasi | West Java | 0.025 | 0.177 | 0.051 |
| 19 | Bandung Barat | West Java | 0.030 | 0.214 | 0.065 |
| 20 | Pangandaran | West Java | 0.038 | 0.231 | 0.071 |
| 21 | Kota Bogor | West Java | 0.029 | 0.195 | 0.058 |
| 22 | Kota Sukabumi | West Java | 0.031 | 0.212 | 0.064 |
| 23 | Kota Bandung | West Java | 0.030 | 0.199 | 0.059 |
| 24 | Kota Cirebon | West Java | 0.031 | 0.201 | 0.060 |
| 25 | Kota Bekasi | West Java | 0.026 | 0.165 | 0.047 |
| 26 | Kota Depok | West Java | 0.027 | 0.176 | 0.051 |
| 27 | Kota Cimahi | West Java | 0.028 | 0.191 | 0.056 |
| 28 | Kota Tasikmalaya | West Java | 0.031 | 0.207 | 0.062 |
| 29 | Kota Banjar | West Java | 0.036 | 0.227 | 0.070 |
| 30 | Cilacap | Central Java | 0.035 | 0.232 | 0.072 |
| 31 | Banyumas | Central Java | 0.036 | 0.236 | 0.073 |
| 32 | Purbalingga | Central Java | 0.035 | 0.233 | 0.072 |
| 33 | Banjarnegara | Central Java | 0.035 | 0.229 | 0.071 |
| 34 | Kebumen | Central Java | 0.037 | 0.247 | 0.078 |
| 35 | Purworejo | Central Java | 0.040 | 0.254 | 0.081 |
| 36 | Wonosobo | Central Java | 0.035 | 0.230 | 0.071 |
| 37 | Magelang | Central Java | 0.036 | 0.236 | 0.073 |
| 38 | Boyolali | Central Java | 0.038 | 0.248 | 0.078 |
| 39 | Klaten | Central Java | 0.039 | 0.249 | 0.079 |
| 40 | Sukoharjo | Central Java | 0.035 | 0.233 | 0.072 |
| 41 | Wonogiri | Central Java | 0.044 | 0.260 | 0.084 |
| 42 | Karanganyar | Central Java | 0.036 | 0.235 | 0.073 |
| 43 | Sragen | Central Java | 0.038 | 0.244 | 0.077 |
| 44 | Grobogan | Central Java | 0.035 | 0.230 | 0.071 |
| 45 | Blora | Central Java | 0.037 | 0.240 | 0.075 |
| 46 | Rembang | Central Java | 0.034 | 0.225 | 0.069 |
| 47 | Pati | Central Java | 0.036 | 0.233 | 0.072 |
| 48 | Kudus | Central Java | 0.031 | 0.206 | 0.062 |
| 49 | Jepara | Central Java | 0.032 | 0.219 | 0.067 |
| 50 | Demak | Central Java | 0.030 | 0.210 | 0.063 |
| 51 | Semarang | Central Java | 0.035 | 0.234 | 0.073 |
| 52 | Temanggung | Central Java | 0.036 | 0.231 | 0.071 |
| 53 | Kendal | Central Java | 0.033 | 0.221 | 0.067 |
| 54 | Batang | Central Java | 0.033 | 0.217 | 0.066 |
| 55 | Pekalongan | Central Java | 0.031 | 0.217 | 0.066 |
| 56 | Pemalang | Central Java | 0.033 | 0.224 | 0.068 |
| 57 | Tegal | Central Java | 0.032 | 0.221 | 0.068 |
| 58 | Brebes | Central Java | 0.032 | 0.222 | 0.068 |
| 59 | Kota Magelang | Central Java | 0.036 | 0.229 | 0.071 |
| 60 | Kota Surakarta | Central Java | 0.033 | 0.220 | 0.067 |
| 61 | Kota Salatiga | Central Java | 0.033 | 0.227 | 0.070 |
| 62 | Kota Semarang | Central Java | 0.030 | 0.205 | 0.061 |
| 63 | Kota Pekalongan | Central Java | 0.030 | 0.202 | 0.060 |
| 64 | Kota Tegal | Central Java | 0.031 | 0.210 | 0.063 |
| 65 | Kulon Progo | Yogyakarta | 0.040 | 0.248 | 0.078 |
| 66 | Bantul | Yogyakarta | 0.035 | 0.232 | 0.072 |
| 67 | Gunung Kidul | Yogyakarta | 0.042 | 0.253 | 0.080 |
| 68 | Sleman | Yogyakarta | 0.033 | 0.223 | 0.068 |
| 69 | Kota Yogyakarta | Yogyakarta | 0.032 | 0.215 | 0.065 |
| 70 | Pacitan | East Java | 0.042 | 0.252 | 0.080 |
| 71 | Ponorogo | East Java | 0.041 | 0.247 | 0.078 |
| 72 | Trenggalek | East Java | 0.039 | 0.240 | 0.075 |
| 73 | Tulungagung | East Java | 0.038 | 0.237 | 0.074 |
| 74 | Blitar | East Java | 0.039 | 0.243 | 0.076 |
| 75 | Kediri | East Java | 0.036 | 0.231 | 0.071 |
| 76 | Malang | East Java | 0.036 | 0.228 | 0.070 |
| 77 | Lumajang | East Java | 0.036 | 0.222 | 0.068 |
| 78 | Jember | East Java | 0.035 | 0.224 | 0.068 |
| 79 | Banyuwangi | East Java | 0.037 | 0.230 | 0.071 |
| 80 | Bondowoso | East Java | 0.038 | 0.227 | 0.070 |
| 81 | Situbondo | East Java | 0.036 | 0.218 | 0.067 |
| 82 | Probolinggo | East Java | 0.034 | 0.217 | 0.066 |
| 83 | Pasuruan | East Java | 0.031 | 0.201 | 0.060 |
| 84 | Sidoarjo | East Java | 0.029 | 0.190 | 0.056 |
| 85 | Mojokerto | East Java | 0.033 | 0.211 | 0.063 |
| 86 | Jombang | East Java | 0.034 | 0.223 | 0.069 |
| 87 | Nganjuk | East Java | 0.037 | 0.231 | 0.072 |
| 88 | Madiun | East Java | 0.041 | 0.241 | 0.075 |
| 89 | Magetan | East Java | 0.043 | 0.251 | 0.080 |
| 90 | Ngawi | East Java | 0.040 | 0.236 | 0.074 |
| 91 | Bojonegoro | East Java | 0.037 | 0.229 | 0.071 |
| 92 | Tuban | East Java | 0.035 | 0.223 | 0.068 |
| 93 | Lamongan | East Java | 0.037 | 0.226 | 0.069 |
| 94 | Gresik | East Java | 0.031 | 0.201 | 0.059 |
| 95 | Bangkalan | East Java | 0.032 | 0.227 | 0.069 |
| 96 | Sampang | East Java | 0.029 | 0.211 | 0.064 |
| 97 | Pamekasan | East Java | 0.031 | 0.211 | 0.064 |
| 98 | Sumenep | East Java | 0.036 | 0.218 | 0.066 |
| 99 | Kota Kediri | East Java | 0.033 | 0.212 | 0.064 |
| 100 | Kota Blitar | East Java | 0.035 | 0.225 | 0.069 |
| 101 | Kota Malang | East Java | 0.031 | 0.211 | 0.064 |
| 102 | Kota Probolinggo | East Java | 0.032 | 0.207 | 0.062 |
| 103 | Kota Pasuruan | East Java | 0.030 | 0.203 | 0.060 |
| 104 | Kota Mojokerto | East Java | 0.033 | 0.209 | 0.063 |
| 105 | Kota Madiun | East Java | 0.037 | 0.227 | 0.070 |
| 106 | Kota Surabaya | East Java | 0.030 | 0.194 | 0.057 |
| 107 | Kota Batu | East Java | 0.034 | 0.221 | 0.068 |
| 108 | Pandeglang | Banten | 0.029 | 0.205 | 0.061 |
| 109 | Lebak | Banten | 0.028 | 0.194 | 0.057 |
| 110 | Tangerang | Banten | 0.025 | 0.172 | 0.049 |
| 111 | Serang | Banten | 0.027 | 0.185 | 0.054 |
| 112 | Kota Tangerang | Banten | 0.026 | 0.161 | 0.046 |
| 113 | Kota Cilegon | Banten | 0.026 | 0.167 | 0.048 |
| 114 | Kota Serang | Banten | 0.025 | 0.167 | 0.048 |
| 115 | Kota Tangerang Selatan | Banten | 0.027 | 0.169 | 0.048 |

**Table S2. Model parameters descriptions and values for the metapopulation model.**

| **Parameter** | **Symbol** | **Value** | **Description** |
| --- | --- | --- | --- |
| Transmission rate | $\beta_{i}$ | - | Calculated from $R_{0,i}$. |
| Basic reproduction number | $R_{0,i}$ | - | Estimated from the maximum values of the smoothing spline models between mobility changes and $R_{t,funerals}$. |
| Relative changes in daily transmission rate | $\kappa_{i}$ | - | The ratio between $R_{0,i}$ and daily estimated $R_{t,i}$ based on spline smoothing models. |
| Mean latent period | $1/\alpha$ | 4.6 days | Estimated as 5.1 days [6] with 0.5 days accounted as a pre-symptomatic period of infectiousness. |
| Mean duration of infectiousness of mild infections | $1/\gamma_{1}$ | 2.1 days | 0.5 days infectiousness period prior to symptoms included which in combination with mean duration of severe illness gives a mean serial interval of 6.75 days [7]. |
| Mean duration of infectiousness of severe infections | $1/\gamma_{2}$ | 4.5 days | Mean onset to admission to hospital of 4 days, as used in squire model [8], based on unpublished analysis of data from the ICNARC study [9] and includes 0.4 days of infectiousness prior to symptoms. |
| Probability of having severe illness, needing hospitalisations | $p_{hosp,i}$ | **Table S1** | For each district, the probability was calculated by running a full unmitigated epidemic in squire package [10] The proportion of people needing hospitalisations were calculated based on the simulations. |
| Probability of needing critical care, if hospitalised | $p_{critical\vert hosp,i}$ | **Table S1** | For each district, the probability was calculated by running a full unmitigated epidemic in squire package [10] The proportion of people needing hospitalisations were calculated based on the simulations. |
| Probability of death of severe illness that does not need critical care | $p_{\begin{aligned} death\vert\\ non critical,i \end{aligned}}$ | **Table S1** | For each district, the probability was calculated by running a full unmitigated epidemic in squire package [10]. The proportion of people needing hospitalisations were calculated based on the simulations. |
| Probability of death of severe illness that needs critical care | $p_{\begin{aligned} death\vert\\ critical,i \end{aligned}}$ | 0.5 | Probability of death from severe illness needing critical care based on the data of the ICNARC study in the UK [9]. |

**Table S3. List of transmission scenarios and counterfactual scenarios for model simulations.**

| **Transmission scenarios** | | |
| --- | --- | --- |
| **Scenario name** | **Details** | **Results shown in** |
| Rural 100% | $R_{0}$ and $R_{t}$ of urban and rural districts in each province were assumed to be the same. | **Figure S5** |
| Rural 90% | $R_{0}$ and $R_{t}$ of rural districts were assumed to be 90% of the province level $R_{0}$ and $R_{t}$. Used as the main transmission scenario shown in the main text. | **Figure 4; Figure S5** |
| Rural 75% | $R_{0}$ and $R_{t}$ of rural districts were assumed to be 75% of the province level $R_{0}$ and $R_{t}$. | **Figure S5** |
| Rural 60% | $R_{0}$ and $R_{t}$ of rural districts were assumed to be 60% of the province level $R_{0}$ and $R_{t}$. | **Figure S5** |

| **Metapopulation simulation scenarios (as shown in Table 1 in the main text)** | | | |
| --- | --- | --- | --- |
| **Scenario name** | **Details** | **Transmission scenario used** | **Results shown in** |
| Baseline | Movement from a district is assumed to reduce according to reductions in movement within a district scaled by an odds ratio of 2 to reflect assumed lower likelihood of long-distance travel. | Rural 90% | **Figure 4; Figure S6 (as a sensitivity analysis comparing to Ramadan mobility scaling with OR=100); Table 2** |
| Ramadan 1 | No movement reductions between districts during the Ramadan and Eid festivals period and the $R_{t}$ values during the period were assumed to be similar to the main/actual scenario. | Rural 90% | **Figure 4** |
| Ramadan 2 | No movement reductions between districts during the Ramadan and Eid festivals period and the $R_{t}$ values during the period were assumed to be 75% of each district $R_{0,i}$. | Rural 90% | **Figure 4** |
| Ramadan 3 | No movement reductions between districts during the Ramadan and Eid festivals period and the $R_{t}$ values during the period were assumed to be the same as each district $R_{0,i}$. | Rural 90% | **Figure 4** |
| Unmitigated | No interventions assumed which implies no movement reductions over all period of simulations with the $R_{t}$ values to be the same as each district $R_{0,i}$ over the period of simulations. | Rural 90% | **Figure 4; Table 2** |

**Table S4. List of future projection scenarios.**

| **Projection scenarios to calculate lives lost and remaining to save** | | |
| --- | --- | --- |
| **Scenario name** | **Details** | **Output** |
| Unmitigated ($R_{c}=2.00$) – **main text** | Based on the model fitted to confirmed and suspected deaths data, forward projections were simulated assuming the $R_{c}=2.00$ from 8^th^ December 2020 onwards. | **Projected number of lives lost and remaining to save (Figure 6)** |
| Current trajectory ($R_{c}=1.25\to2.00$) – **main text** | Based on the model fitted to confirmed and suspected deaths data, forward projections were simulated assuming the $R_{c}=1.25$ (a representative value for current outbreak trajectory) from 8^th^ December 2020 onwards. Transmission then ‘returns to normal’ at the levels earlier in the pandemic ($R_{c}=2.00$), simulated in the beginning of February, March, April, May, and June. | **Projected number of lives lost and remaining to save (Figure 6)** |
| Suppression ($R_{c}=0.75\to2.00$) – **main text** | Based on the model fitted to confirmed and suspected deaths data, forward projections were simulated assuming the $R_{c}=0.75$ (a representative value for a transmission suppression strategy) from 8^th^ December 2020 onwards. Transmission then ‘returns to normal’ at the levels earlier in the pandemic ($R_{c}=2.00$), simulated in the beginning of February, March, April, May, and June. | **Projected number of lives lost and remaining to save (Figure 6)** |
| Unmitigated ($R_{c}=3.00$) – **sensitivity analysis** | Based on the model fitted to confirmed and suspected deaths data, forward projections were simulated assuming the $R_{c}=3.00$ (assuming a higher basic reproduction number of the virues than the main simulation scenarios) from 8^th^ December 2020 onwards. | **Projected number of lives lost and remaining to save (Figure S10)** |
| Current trajectory ($R_{c}=1.25\to3.00$) – **sensitivity analysis** | Based on the model fitted to confirmed and suspected deaths data, forward projections were simulated assuming the $R_{c}=1.25$ (a representative value for current outbreak trajectory) from 8^th^ December 2020 onwards. Transmission then ‘returns to normal’ at the levels earlier in the pandemic ($R_{c}=3.00$), simulated in the beginning of February, March, April, May, and June. | **Projected number of lives lost and remaining to save (Figure S10)** |
| Suppression ($R_{c}=0.75\to3.00$) – **sensitivity analysis** | Based on the model fitted to confirmed and suspected deaths data, forward projections were simulated assuming the $R_{c}=0.75$ (a representative value for a transmission suppression strategy) from 8^th^ December 2020 onwards. Transmission then ‘returns to normal’ at the levels earlier in the pandemic ($R_{c}=3.00$), simulated in the beginning of February, March, April, May, and June. | **Projected number of lives lost and remaining to save (Figure S10)** |

| **Projection scenarios to assess pressure on healthcare capacity** | | |
| --- | --- | --- |
| **Scenario name** | **Details** | **Output** |
| $R_{c}=0.75\to2.00$ (Susp.) | Based on the model fitted to suspected deaths data, forward projections were simulated assuming the $R_{c}$ dropped to 0.75. Transmission returns to levels observed at the beginning of the outbreak (i.e. $R_{c}=2)$ due to behaviour change once burden declines to low levels (total deaths for 7 consecutive days < 7). | **Daily number of deaths (Figure S11)** |
| $R_{c}=0.75\to1.25$ (Susp.) | Based on the model fitted to suspected deaths data, forward projections were simulated assuming the $R_{c}$ dropped to 0.75. Transmission returns to current levels observed during AKB (i.e. $R_{c}=1.25)$ due to behaviour change and sustained intervention policies once burden declines to low levels (total deaths for 7 consecutive days < 7). | **Daily number of deaths (Figure S11)** |
| $R_{c}=0.75\to1.25$ (Conf.) | Based on the model fitted to suspected deaths data, forward projections were simulated assuming the $R_{c}$ dropped to 0.75. Transmission returns to current levels observed during AKB (i.e. $R_{c}=1.25)$ due to behaviour change and sustained intervention policies once burden declines to low levels (total deaths for 7 consecutive days < 7). | **Daily number of deaths (Figure S11)** |
| $R_{c}=1.25\to2.00$ (Susp.) | Based on the model fitted to suspected deaths data, forward projections were simulated assuming the $R_{c}$ stayed at the current estimated level in all provinces (1.25 - within the range of the most recent point estimates in **Figs. S9 & S10**). Transmission returns to levels observed at the beginning of the outbreak (i.e. $R_{c}=2)$ due to behaviour change once burden declines to low levels (total deaths for 7 consecutive days < 7). | **Daily number of deaths and healthcare demand (Figure S11)** |
| $R_{c}=1.25\to2.00$ (Conf.) | Based on the model fitted to confirmed deaths data, forward projections were simulated assuming the  $R_{c}$ stayed at the current estimated level in all provinces (1.25). Transmission returns to levels observed at the beginning of the outbreak (i.e. $R_{c}=2)$ due to behaviour change once burden declines to low levels (total deaths for 7 consecutive days < 7). | **Daily number of deaths and healthcare demand (Figure S11)** |
| $R_{c}=2.00$ (Susp.) | Based on the model fitted to suspected deaths data, forward projections were simulated assuming the $R_{c}$ increased to the initial level of 2.00 in all provinces, representing ‘back-to-normal’ condition. | **Daily number of deaths (Figure S11)** |

**Table S5. Estimated attack rate in each province in Java island based on models fitted to confirmed or suspected deaths data on 2^nd^ September 2020 and 7^th^ December 2020.**

| **Province** | **Deaths data type** | **Estimation date** | **Attack rate in percentage (95% CrI)** |
| --- | --- | --- | --- |
| JAKARTA | Confirmed deaths | 02-Sep | 4.61 (4.22-5.2) |
| JAKARTA | Confirmed deaths | 07-Dec | 9.75 (8.74-11.49) |
| JAKARTA | Suspected deaths | 02-Sep | 18.43 (17.04-19.66) |
| JAKARTA | Suspected deaths | 07-Dec | 32.48 (30.09-34.63) |
| WEST JAVA | Confirmed deaths | 02-Sep | 0.2 (0.17-0.24) |
| WEST JAVA | Confirmed deaths | 07-Dec | 0.66 (0.54-0.75) |
| WEST JAVA | Suspected deaths | 02-Sep | 0.41 (0.36-0.49) |
| WEST JAVA | Suspected deaths | 07-Dec | 1.42 (1.25-1.67) |
| CENTRAL JAVA | Confirmed deaths | 02-Sep | 0.97 (0.85-1.13) |
| CENTRAL JAVA | Confirmed deaths | 07-Dec | 2.3 (1.96-2.81) |
| CENTRAL JAVA | Suspected deaths | 02-Sep | 2.41 (2.05-2.88) |
| CENTRAL JAVA | Suspected deaths | 07-Dec | 5.96 (5.1-6.98) |
| YOGYAKARTA | Confirmed deaths | 02-Sep | 0.41 (0.33-0.49) |
| YOGYAKARTA | Confirmed deaths | 07-Dec | 1.39 (1.05-1.68) |
| YOGYAKARTA | Suspected deaths | 02-Sep | 1.12 (0.92-1.37) |
| YOGYAKARTA | Suspected deaths | 07-Dec | 4.09 (3.33-5.24) |
| EAST JAVA | Confirmed deaths | 02-Sep | 2.13 (1.89-2.33) |
| EAST JAVA | Confirmed deaths | 07-Dec | 3.85 (3.39-4.4) |
| EAST JAVA | Suspected deaths | 02-Sep | 3.35 (2.89-3.83) |
| EAST JAVA | Suspected deaths | 07-Dec | 6.17 (5.46-7.38) |
| BANTEN | Confirmed deaths | 02-Sep | 0.34 (0.29-0.41) |
| BANTEN | Confirmed deaths | 07-Dec | 1.08 (0.9-1.34) |
| BANTEN | Suspected deaths | 02-Sep | 0.59 (0.5-0.68) |
| BANTEN | Suspected deaths | 07-Dec | 2.09 (1.75-2.51) |

**References**

1. WHO Indonesia. COVID-19 Indonesia Situation Reports. 2020. https://www.who.int/indonesia/news/novel-coronavirus/situation-reports.

2. Ministry of Health of the Republic of Indonesia. Ketahanan Kesehatan dalam Menjalani Tatanan Hidup Baru. Jakarta; 2020.

3. Djaafara BA, Whittaker C, Watson OJ, Verity R, Brazeau NF, Oktavia D, et al. Quantifying the dynamics of COVID-19 burden and impact of interventions in Java, Indonesia. medRxiv. 2020;:2020.10.02.20198663. doi:10.1101/2020.10.02.20198663.

4. Jakarta Provincial Health Department. Jakarta COVID-19 Data Monitoring. 2020. https://corona.jakarta.go.id/id/data-pemantauan. Accessed 10 Dec 2020.

5. COVID-19 di Indonesia @kawalcovid19 online spreadsheet (tab: Kasus per Provinsi). 2020. kcov.id/daftarpositif.

6. Lauer SA, Grantz KH, Bi Q, Jones FK, Zheng Q, Meredith HR, et al. The Incubation Period of Coronavirus Disease 2019 (COVID-19) From Publicly Reported Confirmed Cases: Estimation and Application. Ann Intern Med. 2020;172:577–82. doi:10.7326/M20-0504.

7. Bi Q, Wu Y, Mei S, Ye C, Zou X, Zhang Z, et al. Epidemiology and transmission of COVID-19 in 391 cases and 1286 of their close contacts in Shenzhen, China: a retrospective cohort study. Lancet Infect Dis. 2020;0. doi:10.1016/S1473-3099(20)30287-5.

8. Walker PGT, Whittaker C, Watson OJ, Baguelin M, Winskill P, Hamlet A, et al. The impact of COVID-19 and strategies for mitigation and suppression in low- and middle-income countries. Science (80- ). 2020;369:eabc0035. doi:10.1126/science.abc0035.

9. Intensive Care National Audit & Research Centre. ICNARC report on COVID-19 in critical care.

10. Watson OJ, Walker P, Whittaker C, Winskill P, Charles G. squire: SEIR transmission model of COVID-19. https://github.com/mrc-ide/squire.
