# Supplementary material for: Using syndromic measures of mortality to capture the dynamics of COVID-19 in Java, Indonesia, in the context of vaccination rollout
Source: BMC Med. 2021 Jun 18;19:146. doi: 10.1186/s12916-021-02016-2 (PMC8212796; doi:10.1186/s12916-021-02016-2)
Supplement: Supplementary file 2 — Additional file 2. Supplementary methods section of ‘Using syndromic measures of mortality to capture the dynamics of COVID-19 in Java, Indonesia in the context of vaccination rollout’. This additional file comprises of all supplementary methods accompanying methods described in the main text. [file 12916_2021_2016_MOESM2_ESM.docx]

Additional file 2

**S1. Data Sources and Curation**

**Epidemiological data sources for Jakarta**

Epidemiological data for Jakarta were obtained from the official Jakarta COVID-19 data monitoring website (<https://corona.jakarta.go.id/id/data-pemantauan>) [1]. This data comprises daily reported cases, reported deaths, funerals with COVID-19 protocol (C19P), and the number of tests. We collate the data for analysis up to 7^th^ December 2020.

Anonymous individual data of 11,280 confirmed COVID-19 cases up to 29^th^ June 2020 in Jakarta were obtained from the Jakarta Department of Health. Data consist of dates of onset of symptoms, dates of attendance in the hospital, and dates of deaths. The individual data were used to estimate the delay distributions between onset to diagnosis and onset to death.

**Epidemiological data sources for five other provinces in Java (Banten, West Java, Central Java, Yogyakarta, and East Java)**

Daily reported cases and reported deaths data for five other provinces in Java were obtained from an independently curated online spreadsheet by a crowdsource organisation KawalCOVID19 ([kcov.id/daftarpositif](https://t.co/c8OCdRgkWJ?amp=1)) [2] based on the daily publication by Indonesia COVID-19 National Task Force [3] (data for analysis were collated up 7^th^ December 2020). The weekly number of deaths of suspected and probable cases was obtained from WHO Indonesia situation reports 13-36 [4].

**Call detail records data**

Anonymised call detail records (CDRs) data from one of the biggest telecommunication companies in Indonesia over the period of 1^st^ May 2011 to 30^th^ April 2012 were used to estimate between-district movement matrices for the Ramadan and non-Ramadan period. The CDRs data were collected daily with a total of 266 billion records and 137 million unique SIM cards. There was a total of 17,319 mobile phone towers operated during the period of CDRs data collection across the country.

**Province-level mobility changes**

Province-level mobility changes data were acquired from the freely-available Google COVID-19 Community Mobility Reports (<https://www.google.com/covid19/mobility/>) [5]. Google Mobility Reports data up to 7^th^ of December 2020 were used for the analysis.

**Healthcare capacity data**

District-level hospital and ICU beds data were obtained from the Online Healthcare Facilities (*Fasyankes Online*) website by the Directorate General of Health Services (*Ditjen Yankes*) of the Ministry of Health of the Republic of Indonesia [6].

**Dedicated COVID-19 isolation beds and ICU beds data**

Data for the capacity of dedicated COVID-19 isolation beds and ICU beds were obtained from a report from the Ministry of Health in August 2020 [7].

**S2. Reconstruction of frequency of onset**

Daily reported cases, reported deaths, and C19P funerals data in Jakarta were reconstructed to represent the onset day of each reported event using estimates of the distribution of delays between onset and diagnosis and onset and death. Each C19P funeral was assumed to occur the day following the date of death.

The distributions of onset to diagnosis and onset to death of confirmed COVID-19 cases were estimated by fitting discretised Gamma distributions [8] to the individual patient data obtained from the Jakarta Department of Health (Additional file 1: Figure S1). The mean estimate of the onset to diagnosis delay was 7.62 days, with a standard deviation of 7.51 days. The mean estimate of the onset to death delay was 15.87 days, with a standard deviation of 9.34 days. 100 sets of reconstructed daily frequencies of onset of cases, deaths, and funerals were then calculated on the basis of 100 draws for each reported event from these distributions (respectively onset to hospitalisation, onset to death, and onset to funeral). Adjustment for right censoring occurring due to individuals currently with symptoms but have yet to reach outcome was carried out by dividing the inferred onset frequency on a given day by the cumulative probability it would have been observed by the last date within the dataset.

**S3. Effective reproduction number (**$\boldsymbol{R}_{\boldsymbol{t}}$**) calculations based on reconstructed epidemiological data in Jakarta and its relationship with daily mobility changes**

The daily effective reproduction number in Jakarta was estimated using the EpiEstim R package [9, 10] for each reconstructed cases ($R_{t,cases}$), deaths ($R_{t,deaths}$), funerals ($R_{t,funerals}$) data. $R_{t}$ at the beginning of the epidemic was estimated for the period before 2^nd^ March 2020 (the day where the country’s first two cases were announced). Subsequently, $R_{t}$ was estimated over a weekly sliding window, with a mean and standard deviation of serial interval distribution were assumed to be 6.3 and 4.2 days, respectively [11].

1,000 random samples were drawn from the posterior samples of the estimated $R_{t,cases}$, $R_{t,deaths}$, and $R_{t,funerals}$ at each timepoint. Pearson’s correlation coefficients for estimates based on cases, deaths, and funerals data were calculated against the daily average changes in non-residential mobility in Jakarta based on Google mobility estimates. The daily average changes in non-residential mobility are the average of changes of retail and recreation, grocery and pharmacy, parks, transit stations, and workplaces types of mobility to each respective baseline.

The 7-day rolling average changes of non-residential mobility were fitted to 100 posterior samples of the estimated $R_{t,funerals}$ using smoothing spline models. The implementation of the smoothing spline model was done in R software using *smooth.spline* function with four knots. The models were then used to extrapolate the daily values of $R_{t}$ outside of Jakarta based on the province-level 7-day rolling average of changes in non-residential mobility (Google Community Mobility Reports [5]).

**S4. Estimating movement matrices from CDRs data**

District level (city and municipality, 115 in total) movement matrices ($M$) prior to the pandemic for the normal (or non-Ramadan) ($M_{N}$) and Ramadan ($M_{R}$) periods were calculated from CDRs data. Each element of the matrix ($m_{i,j}$) represents the proportion of days spent by residents of district $i$ in district $j$ over the year.

The daily position of each user is described as the district where the most frequent mobile phone usage happened over the period of a single day. All users were assumed to be active from the first day to the last day of their phone usage. On days where the user was not active (no phone activities recorded), the position of the user was assumed to be the same as the previous day. The primary residence of each user is defined as the district where the users spent most of their days over their ‘active’ period. Based on their daily locations and primary residences, we then calculated the proportion of days spent of people from district $i$ in district $j$. $M_{R}$ was estimated using data of August and September 2011 (period of the Ramadan month, Eid celebration, and national holidays period). $M_{N}$ was estimated using the rest of the data that were not used to estimate $M_{R}$ (May 2011 - July 2011 and October 2011 - April 2012 periods).

All districts in the Java island were represented as a single row in each matrix with exceptions for districts in Jakarta province which were represented as an aggregated single ($i=1$). Outside Java movements were represented as a single row ($i=2$). Additional file 1: Table S1 shows a complete list of districts (including Jakarta and outside Java) and each respective index in the movement matrix.

**S5. Metapopulation Model**

**Metapopulation model of COVID-19 spread in Java**

We developed a metapopulation model to simulate the spread of COVID-19 in Java. Each patch in the metapopulation model represents districts ($i=1,2,...,115$) listed in Additional file 1: Table S1. For each patch, stochastic differential equations representing a Susceptible-Exposed-Infected-Recovered (SEIR) model were implemented (overall structure in Additional file 1: Figure S2). The equations are as follows:

$$\frac{{dS}_{i}}{dt}= -new infections_{i}$$

$$\frac{{dE}_{i}}{dt}=new infections_{i}-\alpha E_{i}$$

$$\frac{{dI}_{mild,i}}{dt}=(1-p_{hosp,i}) \alpha E_{i}-\gamma_{1}I_{mild,i}$$

$$\frac{{dI}_{case,i}}{dt}=p_{hosp,i} \alpha E_{i}-\gamma_{2}I_{case,i}$$

$$\frac{dR_{i}}{dt}=\gamma_{1}I_{mild,i} + \gamma_{2}I_{case,i}$$

where $new infections_{i}$ is the number of new infections in each patch based on the stochastic adaptation of the metapopulation transmission model by Keeling et al. [12],$\alpha$ is the per-capita transition rate reflecting the mean duration of latent period, $p_{hosp}$ is the probability of having severe illness and needing hospitalisations, $p_{critical|hosp}$ is the probability of needing critical care if hospitalised, and $\gamma_{1}$ and $\gamma_{2}$ are the per-capita transition rate reflecting the mean duration of infectiousness of mild and severe infections, respectively. The full model parameter descriptions and specifications are available in Additional file 1: Table S2. To calculate $new infections_{i}$, we firstly need to calculate the district-level force of infections $\lambda_{i}$ that accounts for inter-district movements of both susceptible individuals (that might acquire infections in other districts) and infected individuals (that might infect people in other districts) based on the inter-patch connectivities (the movement matrix, $M$, accounting daily changes in mobility - see **section S4**). The total number of infected individuals that are contributing to infections in district $i$, $I_{tot,i}$, is calculated by:

$I_{tot,i}=\sum_{j=1}^{115} Binomial(I_{j},m_{j,i})$.

The transmission rate for each district is calculated by:

$\beta_{i}=\frac{R_{0,i}}{\left( (1-p_{hosp,i})\gamma_{1}+p_{hosp,i}\gamma_{2} \right)}$,

where $R_{0,i}$ is the value of $R_{t}$estimated for the period of maximum mobility recorded within Google Mobility data.

Hence,:

$\lambda_{i}=\beta_{i}\times\kappa_{t,i}\times\frac{I_{tot,i}}{N_{i}}$,

where $N_{i}$ is the total population of each district/patch and $\kappa_{t,i}$ is the daily ratio between the estimated $R_{t,i}$ values based on the spline model estimates and the respective $R_{0,i}$, representing the relative changes in the daily transmission rate. We assumed no transmission contributed to and from outside Java but we still allow movement to and from that patch ($i=2$) which implies both $I_{2}$ and $\lambda_{2}$ are always 0.

$new infections_{i}$ are then calculated as:

$${new infections}_{i}=\sum_{j=1}^{115} Binomial(S_{i}\times m_{i,j},(1-exp(-\lambda_{j})))$$

For each severe infection needing hospitalisation ($I_{case}$), the case was either deemed a critical case (i.e., indicated to require an ICU bed) with probability $p_{critical|hosp}$ and otherwise, non-critical (i.e., indicated to require an isolation bed) with associated probability of death ($p_{death|non critical}$ and $p_{death|critical}$, diagram in Additional file 1: Figure S2B). $p_{hosp}$, $p_{critical|hosp}$, $p_{death|non critical}$ and $p_{death|critical}$ were obtained for each district as the average values estimated within simulations from the squire package [13], taking age-specific demography using district-level census data and, in the absence of equivalent data from Java, mixing patterns based upon a contact survey from Shanghai province, China as an example of contact patterns within a UMIC Asian country and province containing a megacity.

The model was simulated with 100 replicates by seeding initial cases in Jakarta, and Kota Surabaya (East Java’s capital) – both have international airports receiving travellers from China – on 7^th^ January 2020 (arbitrarily selected) with 100 replicates. Initial cases in Jakarta were set to 12, obtained through calibration to provide simulated deaths trends bounded by the interval between reported deaths and C19P funerals. Initial cases in Surabaya were drawn according to a binomial draw assuming an underlying importation rate of 60% of that in Jakarta [14], random numbers based on the binomial distribution were sampled for each replicate.

We assumed different transmission scenarios of districts classified as rural and urban districts. We classified an urban/rural status to each district based upon the urban/rural classification of the majority of villages within the district. We assigned $R_{t,i}$ values for all districts to be the province-level value of $R_{t,i}$ in which each district is located. We then explore the possibilities of rural districts to have different $R_{t,i}$ levels, ranging from 100% to 60% of province-level $R_{t,i}$. In the results section of the main text, we show simulation results assuming $R_{t,i}$ in rural districts to be 90% of $R_{t,i}$ in urban districts. We also ran the model for several different counterfactual scenarios. The list of transmission scenarios and the counterfactual scenarios were shown in Additional file 1: Table S3.

During the period of Ramadan and Eid festivals, 24^th^ April 2020 up to 7^th^ June 2020, the Ramadan movement matrix ($M_{R}$) was used as the baseline movement matrix. In the other period, the non-Ramadan movement matrix ($M_{N}$) was used as the baseline movement matrix. As a baseline assumption, throughout the simulation, the proportions of people spending their time in other districts ($m_{i,j}$ where $i\neq j$) were adjusted by the province-level changes in mobility over time, with reductions in larger-scale movement outside the province assumed to be higher than those within the province according to an odds ratio (OR) of 2 within our default scenario.

For each scenario, we also devised a metric to assess the extent to which the epidemic would be likely to strain available healthcare resources over time given patterns of spatial spread and disparities in healthcare supply by district. This metric was defined by determining the number of available beds for each individual newly requiring hospitalisation each day within the model by subtracting the number of hospital beds required in the simulation from the total hospital beds capacity available at the district-level obtained from the Online Healthcare Facilities website [6].

**S6. Model fitting to confirmed and suspected COVID-19 deaths and future projection scenarios in all provinces in Java**

**Estimating the number of deaths from suspected and probable cases in Java provinces**

Jakarta reported a time series dataset of the province daily C19P funerals in their official COVID-19 tracker website (<https://corona.jakarta.go.id/id/data-pemantauan>) [1] which includes confirmed/reported and probable COVID-19 deaths (both combined were then defined as suspected deaths). Whilst for the other five provinces in Java, daily time series data of probable deaths are not available. WHO Indonesia situation reports provide a weekly summary of confirmed and probable deaths (which both combined become suspected deaths) in all provinces in Java since the end of May 2020 (Additional file 1: Figure S7)[4] We collated these data and calculated the proportion of confirmed deaths from suspected COVID-19 deaths for each province ($\rho_{i}$ with $i$ as each province index).

For all days from 1^st^ March 2020, up to 7^th^ December 2020, we simulated the number of probable deaths in five Java provinces other than Jakarta. Firstly, we aggregated the daily confirmed deaths in each province to a weekly period ($D_{i,t}$ with $t$ as the weekly time window). For each weekly time window $t$, using Negative Binomial distribution, we simulated the number of probable deaths ($O_{i,t}$) in each province 10 times:

$O_{i,t}=NB(D_{i,t},\rho_{i})$.

For each simulated $O_{i,t}$, we simulated the spread of the weekly total estimated probable deaths into daily estimated probable deaths using Multinomial distribution, assuming equal probability for all days during the week 10 times:

$$o_{i,t}=Multinom(O_{i,t},\pi)$$

where $\pi$ is a vector of length 7, where each value is 1/7.

The simulations resulted in 100 samples of estimated daily probable deaths in each province. Adding the simulated daily probable deaths to the daily confirmed deaths, we obtained 100 samples of the estimated number of daily suspected COVID-19 deaths in five provinces in Java other than Jakarta.

The daily suspected COVID-19 deaths are then defined, for Jakarta, as the daily C19P funerals, and for five other Java provinces as the median of the estimated number of the daily suspected COVID-19 deaths.

**Model fitting**

Using the framework developed in the Imperial College COVID-19 LMIC reports [15], we fit the model to both the daily COVID-19 confirmed deaths and the daily COVID-19 suspected deaths data for each province in Java, estimating both a province-specific $R_{0}$ and epidemic start date. To provide model fits that are agnostic to the mobility profiles in each province, we model the time-varying reproduction number, $R_{t}$, using a series of pseudo-random walk parameters, which alter the transmission every 2-weeks, with $R_{t}$ given by:

$$R_{t} = R_{0} . f({-\rho}_{1}-\rho_{2} ... -\rho_{n})$$

Where $f(x) = 2 exp(x)/(1 + exp(x))$, i.e., twice the inverse logit function, which has been used in previous models to capture the impact of mobility data on transmission [16]. Each $\rho$ parameter is introduced two weeks after the previous parameter, serving to capture changes in transmission over time. More specifically, each $\rho$ parameter is set equal to 0 for each day prior to its start date. For example, $\rho_{1}$ is the change in transmission, which is set to start at the beginning of the epidemic. The estimated value for $\rho_{1}$ is then maintained for all future time points. $\rho_{2}$ is the second change in transmission, which starts 14 days after the epidemic start date, i.e., is equal to 0 prior to this. The last mobility independent change in transmission, $\rho_{n}$ is maintained for the last 4 weeks prior to the current day to reflect our inability to estimate the effect size of this parameter due to the approximate 21 day delay between infection and death [17].

The model fitting results were presented in Additional file 1: Figure S8 & S9. Based on the fitted models in all provinces considering different types of deaths data, we estimated the attack rate at the province level and Java level.

**Future projection scenarios**

Using the fitted models, some future scenarios were explored based on the assumed values of the reproduction number under control, $R_{c}$, defined similarly to $R_{0}$ as the average number of secondary infections within an entirely susceptible population but incorporating the impact of NPIs (and, equivalently, $R_{t}$ but not incorporating the effects of population-level immunity such that $R_{0}>R_{c}>R_{t}$). Moreover, as with$R_{0}, R_{c}$>1 can lead to $R_{t}<1$ and a declining epidemic if there exist sufficient levels of naturally acquired immunity within the population. We simulated forward projections based on scenarios described in Additional file 1: Table S4.

**References**

1. Jakarta Provincial Health Department. Jakarta COVID-19 Data Monitoring. 2020. https://corona.jakarta.go.id/id/data-pemantauan. Accessed 10 Dec 2020.

2. COVID-19 di Indonesia @kawalcovid19 online spreadsheet (tab: Kasus per Provinsi). 2020. kcov.id/daftarpositif.

3. Satuan Tugas Penanganan COVID-19 (Indonesia COVID-19 Response Acceleration Task Force). Peta Sebaran. 2020. https://covid19.go.id/peta-sebaran. Accessed 3 Feb 2021.

4. WHO Indonesia. COVID-19 Indonesia Situation Reports. 2020. https://www.who.int/indonesia/news/novel-coronavirus/situation-reports.

5. Google LLC. Google COVID-19 Community Mobility Reports. https://www.google.com/covid19/mobility/. Accessed 22 Aug 2020.

6. Directorate General of Health Services Ministry of Health of the Republic of Indonesia. Fasyankes Online. http://sirs.yankes.kemkes.go.id/fo/. Accessed 11 Mar 2020.

7. Ministry of Health of the Republic of Indonesia. Ketahanan Kesehatan dalam Menjalani Tatanan Hidup Baru. Jakarta; 2020.

8. Chakraborty S, Chakravarty D. Discrete gamma distributions: Properties and parameter estimations. Commun Stat - Theory Methods. 2012;41:3301–24. doi:10.1080/03610926.2011.563014.

9. Cori A, Ferguson NM, Fraser C, Cauchemez S. A new framework and software to estimate time-varying reproduction numbers during epidemics. Am J Epidemiol. 2013;178:1505–12. doi:10.1093/aje/kwt133.

10. Cori A. EpiEstim: A Package to Estimate Time Varying Reproduction Numbers from Epidemic Curves. R package version 2.2-3. 2020. https://cran.r-project.org/package=EpiEstim.

11. Bi Q, Wu Y, Mei S, Ye C, Zou X, Zhang Z, et al. Epidemiology and transmission of COVID-19 in 391 cases and 1286 of their close contacts in Shenzhen, China: a retrospective cohort study. Lancet Infect Dis. 2020;0. doi:10.1016/S1473-3099(20)30287-5.

12. Keeling MJ, Danon L, Vernon MC, House TA. Individual identity and movement networks for disease metapopulations. Proc Natl Acad Sci U S A. 2010;107:8866–70. doi:10.1073/pnas.1000416107.

13. Watson OJ, Walker P, Whittaker C, Winskill P, Charles G. squire: SEIR transmission model of COVID-19. https://github.com/mrc-ide/squire.

14. WorldPop. Global Flight Data Annual. 2020. doi:10.5258/SOTON/WP00100.

15. Imperial College COVID-19 LMIC Reports. Version 5. MRC Centre for Global Infectious Disease Analysis, Imperial College London. 2020. https://mrc-ide.github.io/global-lmic-reports/.

16. Unwin HJT, Mishra S, Bradley VC, Gandy A, Mellan TA, Coupland H, et al. State-level tracking of COVID-19 in the United States. medRxiv. 2020;2 May:2020.07.13.20152355. doi:10.1101/2020.07.13.20152355.

17. Walker PGT, Whittaker C, Watson OJ, Baguelin M, Winskill P, Hamlet A, et al. The impact of COVID-19 and strategies for mitigation and suppression in low- and middle-income countries. Science (80- ). 2020;369:eabc0035. doi:10.1126/science.abc0035.
